# Glutamatergic Circuits in the Pedunculopontine Nucleus Modulate Multiple Motor Functions

**DOI:** 10.1007/s12264-024-01314-y

**Published:** 2024-11-11

**Authors:** Yanwang Huang, Shangyi Wang, Qingxiu Wang, Chaowen Zheng, Feng Yang, Lei Wei, Xintong Zhou, Zuoren Wang

**Affiliations:** 1 Institute of Neuroscience, State Key Laboratory of Neuroscience, CAS Center for Excellence in Brain Science and Intelligence Technology, Chinese Academy of Sciences, Shanghai, 200031 China; 2 University of Chinese Academy of Sciences, Beijing, 100049 China; 3 School of Future Technology, University of Chinese Academy of Sciences, Beijing, 100049 China; 4 School of Life Science and Technology, ShanghaiTech University, Shanghai, 201210 China

**Keywords:** Pedunculopontine nucleus, Glutamatergic neuron, Pause-and-play, Caudal pontine reticular nucleus, Anterior gigantocellular reticular nucleus, Zona incerta

## Abstract

The functional role of glutamatergic (vGluT2) neurons in the pedunculopontine nucleus (PPN) in modulating motor activity remains controversial. Here, we demonstrated that the activity of vGluT2 neurons in the rostral PPN is correlated with locomotion and ipsilateral head-turning. Beyond these motor functions, we found that these rostral PPN-vGluT2 neurons remarkably respond to salient stimuli. Furthermore, we systematically traced the upstream and downstream projections of these neurons and identified two downstream projections from these neurons to the caudal pontine reticular nucleus/anterior gigantocellular reticular nucleus (PnC/GiA) and the zona incerta (ZI). Our findings indicate that the projections to the PnC/GiA inhibit movement, consistent with ‘pause-and-play’ behavior, whereas those to the ZI promote locomotion, and others respond to a new ‘pause-switch-play’ pattern. Collectively, these findings elucidate the multifaceted influence of the PPN on motor functions and provide a robust theoretical framework for understanding its physiological and potential therapeutic implications.

**Supplementary Information:**

The online version contains supplementary material available at 10.1007/s12264-024-01314-y.

## Introduction

Motor modulation proficiency is crucial for animals to adapt and thrive in ever-changing environments. In vertebrates, the capacity to adjust movement is enabled by the complex and evolutionarily conserved coordination of the central nervous system; this includes the motor cortex, basal ganglia, thalamus, midbrain, brainstem, and spinal cord. Impairment in any of these interconnected circuits can lead to compromised motor control. Parkinson’s disease (PD), a progressive neurological disorder typified by the degeneration of dopamine neurons in the substantia nigra (SN), manifests symptoms such as tremors, rigidity, bradykinesia, and postural instability [1]. Consequently, improving the quality of life for individuals with PD has driven researchers to explore and implement treatment strategies targeting specific motor-related neural structures and pathways.

The mesencephalic locomotor region (MLR), a functional area bridging the midbrain and the pons, mostly comprises two distinct nuclei: the dorsal caudal cuneiform nucleus (CnF) and the ventral rostral pedunculopontine nucleus (PPN). Early studies applied electrical microstimulation of the MLR to evoke locomotor behaviors such as walking and running in decerebrate cats [2], with subsequent research confirming these effects in a range of species including rats and monkeys, among other species [3]. More recently, the PPN has emerged as a significant target for deep brain stimulation (DBS) in the treatment of advanced PD. Although its efficacy in enhancing locomotion remains a subject of debate, PPN-DBS has been generally reported to mitigate gait freezing and falls in affected patients [4, 5].

The PPN is a reticular nucleus comprising glutamatergic (vGluT2), cholinergic (ChAT), and GABAergic neurons [6–9], each contributing distinctively to motor modulation [10]. However, findings related to the activation of PPN-vGluT2 neurons are notably inconsistent, with studies indicating promotion [11, 12], inhibition [13, 14], or a combination of both effects [15]. It has been proposed that the variability in these outcomes may stem from the manipulation of different subregions within the PPN, which are correlated with the heterogeneous distribution of cell-type-specific neurons. Recently, two studies have shown that activation of either Rbp4^+^ or Chx10^+^ populations, which are subtypes of vGluT2 neurons predominantly found in the rostral region of the PPN, arrest locomotion in mice [16, 17]. Notably, activation of PPN-vGluT2 Chx10^+^ neurons induces a ‘pause-and-play’ pattern of motor arrest behavior [17].

In addition, the PPN-vGluT2 neurons are known to form extensive connections with various brain regions [11, 14, 18], yet functional investigations have been limited to a select few. For instance, PPN-vGluT2 projections to the basal forebrain [18, 19], lateral hypothalamus [18], horizontal limb of the diagonal band of Broca [15], and the SN [18] have been implicated in the promotion of locomotion. Moreover, optogenetic activation of the projections from Rbp4^+^ MLR-vGluT2 neurons to the SN has been shown to halt locomotion [16]. Of note, researchers have reported that these SN-projecting Rbp4^+^ neurons are primarily associated with the modulation of forelimb movement, such as grooming [16]. Therefore, a comprehensive understanding of the physiological role and circuit mechanisms of PPN-vGluT2 neurons in motor termination warrants further investigation.

In this study, we investigated the physiological roles of vGluT2 neurons in the rostral PPN (rPPN-vGluT2 neurons) in freely-moving rats and revealed that the activity of these neurons is correlated with locomotion and ipsilateral head-turning. Notably, we found that rather than the previously reported ‘pause-and-play’ the motor pattern is switched after the stimulation of rPPN-vGluT2 neurons is turned off. In addition to these roles in locomotor modulation, we also found that rPPN-vGluT2 neurons respond remarkably to salient environmental stimuli. Furthermore, we systematically traced the upstream and downstream projections of those neurons and identified two downstream projections to the caudal pontine reticular nucleus/anterior gigantocellular reticular nucleus (PnC/GiA) and the zona incerta (ZI), respectively. We found that projections to the PnC/GiA stop movement and are responsible for ‘pause-and-play’, while projections to the ZI facilitate locomotion. In addition, we also found that global activation of rostralPPN-vGluT2 neurons also responds to a new ‘pause-switch-play’ pattern of motor modulation.

## Materials and Methods

### Animals

Sprague–Dawley rats were from SLAC Laboratory Animal Co., Ltd. (Shanghai). The *Vglut2*-iCre line was customized by Biocytogen Co., Ltd (Beijing). Rats were housed in pairs and maintained on a 12 h/12 h light/dark schedule at a room temperature of 22–25 °C with water and food *ad libitum* unless otherwise noted. All experimental procedures were approved by the Institutional Animal Care and Use Committee of the Center for Excellence in Brain Science & Intelligence Technology, Chinese Academy of Sciences, Shanghai, China.

### Virus Injection and Stereotaxic Surgery

Male (~8 weeks) and female (~10 weeks) rats were anesthetized using 2.5% isoflurane delivered through an R540 animal anesthesia machine (RWD, Shenzhen, China). Viral vectors were precisely introduced into targeted brain nuclei using a stereotaxic apparatus (Stoelting Co., USA). The coordinates for craniotomy—anteroposterior (AP) from bregma, mediolateral (ML) from the midline, and dorsoventral (DV) from the brain’s surface—were determined based on the sixth edition of Paxinos and Watson’s "The Rat Brain in Stereotaxic Coordinates." Adeno-associated viruses (AAVs) were sourced from Taitool Biotech Co. (Shanghai, China), while the rabies virus was obtained from BrainVTA Co. (Wuhan, China). Throughout the surgical procedures, the rat’s body temperature was regulated with a heating blanket. In addition, to mitigate the risk of pos*t-*surgical infection, the animals were given gentamicin (5 mg/kg, intraperitoneally [i.p.]) and dexamethasone (1 mg/kg, i.p.). A minimum three-week postoperative period was observed to ensure viral expression before subsequent experiments unless otherwise noted.

For fiber photometry recordings, we injected 300 nL of AAV2/8-hSyn-FLEX-GCaMP6s (5.12 × 10^12^ VG/mL) into the PPN (AP 7.6, ML ±1.8, DV 6.4) at 20 nL/min using a UMP3 pump coupled with a SYS-Micro4 controller (World Precision Instruments, USA). To prevent viral leakage, the glass pipette remained in place for 8 min before injection and retraction. We then implanted optical fibers (200 μm OD, 0.37 NA) 0.2 mm over the viral injection site.

For optogenetic stimulation, rats received 300 nL of either AAV2/8-hEF1α-DIO-hChR2-mCherry (3.04 × 10^12^ VG/mL) or AAV2/8-hEF1α-DIO-mCherry (2.96 × 10^12^ VG/mL) into the PPN at the same coordinates. Optical fibers (200 μm OD, 0.37 NA) were implanted 0.5 mm over the virus injection site.

For optogenetic stimulation and extracellular recording, rats were injected with 300 nL of AAV2/8-hEF1α-DIO-hChR2-mCherry (3.04 × 10^12^ VG/mL) into the PPN. Following a 2-week expression period, custom optrodes were implanted. Notably, we added a stainless-steel sleeve to guide the electrode interface board and threaded the ground wire (132 μm stainless steel) through protective tubing. Prior to implantation, tetrodes were gold-plated to adjust impedance to 300–500 kΩ (1 kHz, IMP-2, Bak Electronics Inc., USA). Animals were given a minimum of 7 days to recover from optrode implantation surgery before commencing recordings.

For optogenetic stimulation of PPN vGluT2 neuron axon terminals, we bilaterally injected 300 nL of AAV2/8-hEF1α-DIO-ChR2-mCherry into the PPN and implanted optical fibers 0.2 mm over the terminal nuclei of several brain regions, including the globus pallidus externa (GPe; AP 1.2, ML ± 3.4, DV-5 6), subthalamic nucleus (STN; AP 3.6, ML ± 2.5, DV 7.0), substantia nigra pars reticularis (SNr; (AP 5.4, ML ± 2.8, DV 6.5), PnC/GiA (AP-10.32, ML ± 0.6, DV-8.4), and ZI (AP-2.6, ML ± 1.9, DV-6.8).

To trace anterograde outputs from PPN-vGluT2 neurons, we administered 200 nL of AAV2/5-hSyn-FLEX-tdTomato-T2A-Synaptophysin-EGFP-WPRE-pA (2.34 × 10^12 VG/mL) into the PPN. After a 3-week expression period, rats were euthanized and prepared for immunohistochemistry.

For monosynaptic retrograde tracing inputs of PPN vGluT2 neurons, vGluT2-iCre rats received 200 nL of a helper virus, AAV2/9-hSyn-FLEX-mCherry-2A-TVA-2A-RvG-WPRE-pA (2.29 × 10^12 VG/mL), injected into the PPN. Two weeks later, 150 nL of RV-ENVA-ΔG-EGFP (3 × 10^8^ IFU/mL) rabies virus was administered at the same site. Rats were euthanized for brain sample collection one week pos*t-*injection of the rabies virus.

To selectively stimulate PPN-vGluT2 neurons projecting to the PnC/GiA or ZI, we bilaterally administered 400 nL of AAV2/2Retro-plus-Syn-FLEX-flop (5.03 × 10^12^ VG/mL) into the target region and 300 nL of AAV2/9-hEF1α-fDIO-hChR2-mCherry into the PPN. Optical fibers were implanted 0.5 mm over the PPN virus injection site.

Finally, to dissect the distribution of PPN-vGluT2 neurons projecting to the PnC/GiA and ZI, we respectively injected AAV2/2Retro-EF1α-DIO-H2B-tdTomato and AAV2/2Retro-EF1α-DIO-H2B-EGFP into these regions. Rats were anesthetized and euthanized for brain sample collection after a 3-week expression period.

### Fibre Photometry Recording, Behavioral Assays, and Analysis

For photometry in free-moving animals, optical fibers were interfaced with a triple-color, multi-channel fiber photometry system (ThinkerTech) with 405-nm and 470-nm LED sources. The emission intensity of the 405-nm excitation at the fiber’s tip was finely tuned to ~20 µW, and the 470-nm excitation was adjusted to ~30 µW. Both emission signals and behavioral videos were concurrently recorded at a sampling rate of 30 Hz and time-stamped with the computer’s real-time clock to facilitate synchronization of the Ca^2+^ signals with corresponding animal behavior in subsequent data analysis. To track velocity and head orientation, the behavioral videos were processed and quantified using the graphical user interface of DeepLabCut.

For data analysis, Ca^2+^ signaling was quantified using custom-written MatLab scripts. Initially, photobleaching artifacts were corrected in the raw fluorescence data acquired from both 470-nm and 405-nm channels. Subsequently, the 470-nm channel data underwent a secondary correction using the 405-nm channel as a reference. The relative changes in the Ca^2+^ signal (ΔF/F) were then computed using the formula (F - F0)/F0, where F represents the fluorescence intensity at a given time point and F0 denotes the baseline fluorescence intensity.

To elucidate the relationship between ΔF/F signals and both velocity and head-turning, we conducted a cross-correlation (xcorr) analysis. To assess the correlation between the Ca^2+^ signal and velocity, we calculated the z-scores for both the Ca^2+^ signal and the velocity using a bin size of 100 ms, applying the xcorr function in MatLab (M2021a). To identify head-turning events in rats, we tracked their head movements using DeepLabCut and defined an event as a head turn with an angular velocity >0.01 rad/s. We further categorized each event within a series of binned time windows: '1' for time windows containing a left or right head-turning event and '0' for time windows without any head-turning event. This binary labeling facilitated the identification of head-turning event data. In a similar manner, we calculated the z-scores for both the Ca^2+^ signal and the left or right head-turning events. This methodical approach ensured a systematic investigation of the temporal associations between neural activity, as reflected by ΔF/F signals, and the corresponding behavioral responses.

To clarify the relationship between the activity of rPPN-vGluT2 neurons and salient stimuli, we recorded fiber photometry in response to tactile stimulation by hand or abrupt auditory stimulation. Rats were allowed to move freely within a clear enclosure measuring 40 cm × 30 cm × 60 cm (LWD). For tactile stimulation, a gloved hand touched a rat through the open top of the chamber at intervals of 2 to 3 min, with immediate withdrawal after contact. For auditory stimulation, a 100-ms burst of sound at 100 dB was delivered once per minute. Peri-event time histograms of the Ca^2+^ signal were generated and analyzed in synchronization with the onset of the salient stimuli using MatLab (M2021a).

### Optogenetic Stimulation, Behavioral Assays, and Analysis

For locomotor speed analysis, rats were allowed to move in a matte black open-field chamber (160 cm × 40 cm × 60 cm, LWD). Optogenetic stimulation and recording of a rat’s position and instantaneous velocity were controlled and synchronized by an Anilab instrument and software (Anilab Instrument Co, Ningbo, China). For each stimulation condition, a rat underwent 30−50 repeated trials with a 15−30 s intertrial interval. The width of the laser pulse (473 nm) was 20 ms, and the laser power at the tip of the optic fiber was ~15 mW, as measured by a light intensity meter (Thorlabs, Inc., USA) in all optogenetic experiments. We eliminated trials with speeds <3 cm/s in 500 ms before laser onset, analyzed the speed in 100-ms bins and then calculated the mean velocity changes before, during, and after the stimulus timepoint for all active trials.

For locomotion and motion analysis, rats freely moved in a transparent chamber (40 cm × 30 cm × 60 cm, LWD). Optogenetic stimulation was delivered by a 473-nm laser controlled by Anilab software. The locomotion and motion of rats were recorded by an industrial camera. Behaviors including but not limited to walking, grooming, eating, rearing, balancing on a rod, and swimming were recorded and analyzed.

For respiration rate analysis, anesthetized rats were head-fixed. An air pressure sensor was placed near one of the rat’s nostrils, not touching the nose itself, and the signals from rhythmic nasal breathing were recorded as a measure of respiration. Rats underwent 90 repeated trials with 4 s of optogenetic stimulation and a 26-s intertrial interval. The average normalized respiration rates of all rats were plotted against the time of optogenetic stimulation.

### Brain Slice Recording

Rats injected with AAV2/8-EF1a-DIO-ChR2-mCherry virus were first tested to confirm that their locomotion was halted during optogenetic stimulation; they were then used for brain slice recording. Briefly, rats were anesthetized and perfused transcardially with ice-cold, oxygenated (95% O_2_ and 5% CO_2_) cutting solution (~80 mL) containing the following (in mmol/L): 105 N-methyl-D-glutamine, 105 NaCl, 2.5 KCl, 1.2 NaH_2_PO_4_, 26 NaHCO_3_, 25 glucose, 10 MgSO_4_, 0.5 CaCl_2_, 5 L-ascorbic acid, 3 sodium pyruvate, and 2 thiourea (pH 7.4, 295–305 mOsm). The brain was removed quickly and placed into an ice-cold, oxygenated cutting solution. Coronal slices (350 μm) were prepared using a vibratome (1200 s, Lecia, Germany) and then transferred to an incubation chamber at 32 °C with an oxygenated cutting solution and incubated for 12 min. Then, the slices were transferred into and incubated in artificial cerebrospinal fluid (aCSF) that contained the following (in mmol/L): 119 NaCl, 2.3 KCl, 1.0 NaH_2_PO_4_, 26 NaHCO_3_, 11 glucose, 1.3 MgSO_4_, 2.5 CaCl_2_ (pH 7.4 with HCl, 295–305 mOsm) at room temperature for at least 1 h.

After 1 h of recovery, the slices were transferred to a recording chamber under a microscope (Nikon-FN1) and continuously perfused (1 mL/min) with O_2_-saturated aCSF at 31 °C. Guided by the 520-nm fluorescence signals, we identified and selected PPN neurons with ChR2-mCherry expression for whole-cell patch-clamp recording. Excitation of ChR2 was achieved by 470-nm laser stimulation (25-ms-wide laser pulses, 20 Hz) and controlled by a shutter (Uniblitz, USA). Electrophysiological data were acquired using a MultiClamp 700B amplifier (Axon Instruments, USA). Borosilicate glass electrodes (tip resistance, 4−6 MΩ) were prepared using a Model P-97 Flaming/Brown micropipette puller (Sutter Instruments) and were filled with an internal solution containing (in mmol/L) 126 K-gluconate, 2 KCl, 10 HEPES, 2 MgCl_2_, 4 Na_2_ATP, 0.4 Na_3_GTP, 0.2 EGTA, and 10 creatine phosphate (pH 7.2 with KOH, 290 mOsm) for slice recording.

### Multichannel Electrophysiological Recording and Analysis

Neural signals were recorded (digitized at 40 kHz) and bandpass filtered (300–8000 Hz) using an Omniplex data acquisition system (Plexon Inc., USA). Single units were isolated offline by MClust 3.5 software (A.D. Redish *et al*.; available at http://redishlab.neuroscience.umn.edu/). Single units with a refractory period ≥2 ms and a stable waveform and firing rate during recording were selected. Peri-event time histograms time-locked to laser onset were analyzed and plotted with NeuroExplorer (Version 4.126, Nex Technologies). We calculated the average firing rate of units (using 0.02 ms time bins) for 500 trials of 10 ms and 80 trials of 4-s laser stimulation to identify neurons expressing ChR2 and test the activity induced by laser stimulation.

### Histology

For immunostaining, rats were sacrificed under anesthesia and transcardially perfused with PBS followed by 4% paraformaldehyde (PFA). Brains were postfixed in 4% PFA for 4 h at 4 °C and then transferred to 30% sucrose until sinking to the bottom. Coronal or sagittal sections (30 μm) were cut on a cryostat (CM1950, Leica). Goat anti-ChAT (1:1000, AB144P, Merck Millipore), rabbit anti-TH (1:1000, P40101, Pel-Freez), rabbit anti-RFP (1:1000, 600-401-379, Rockland), rabbit anti-GFP (1:1000, A11122, Thermo Fisher Scientific), and guinea pig anti-c-fos (1:1000, 226 004, Synaptic System) were used as primary antibodies, and all secondary antibodies originated from donkeys (1:500; Invitrogen). After secondary antibody incubation, sections were coverslipped using Fluomoun*t-*G mounting medium with DAPI (Southern Biotech).

For fluorescence *in situ* hybridization and immunostaining, rats were perfused with DEPC-treated PBS. Sections were serially cut (20 μm) and mounted on glass slides, which were stored at − 80 ℃ until the start of the multiplex fluorescent RNAscope assay (323100, ACD Biotech). RNA hybridization probes included antisense probes against genes encoding rat vGluT2 (317011-C1), CaMKIIα (317081-C2), GAD2 (435801-C3), and iCre-C2 (423321-C2). After *in situ* hybridization was finished, immunostaining with anti-ChAT (1:300) or anti-RFP (1:300) was applied. Finally, the slides were coverslipped with Fluomoun*t-*G mounting medium with DAPI. It’s worth noting that, during the RNAscope process, the sample slides were pre-treated with hydrogen peroxide and proteinase K III, and the fluorescent protein lost its original laser excitation ability.

In order to confirm the implantation position of the optical fiber and optrodes, we electrolytically lesioned (25 μA, 20 s) the brains with a stimulus isolator (ISO-Flex, AMPI, Israel) before perfusion. Serial sections (50 μm) were cut and coverslipped with Fluomoun*t-*G mounting medium with DAPI.

For anterograde and monosynaptic retrograde tracing experiments, 30-μm sections were collected at 240-μm intervals. It should be emphasized that the sections required for cell counting in the PPN subregion were collected extremely carefully to precisely match the rat brain atlas as much as possible by adjusting the angle of the blade while sectioning the thalamus, hippocampus, and midbrain. To assess the injection site of AAV2/5-syn-Flex-Tdtomato-P2A-Synaptophysin-EGFP for an anterograde tracking experiment, slides were pre-treated with hydrogen peroxide to eliminate the TdTomato signal and were used for immunostaining with anti-GFP and anti-ChaT (Fig. S8).

Images were captured with an Olympus VS120 or Olympus FV3000 IX83 confocal microscope (Olympus Co., Japan).

### Cell Counting

We experimentally defined the PPN region by combining the expression profile of ChAT and the outline of the PPN in the rat brain atlas and then counted the cells in that region. Coronal sections covering the PPN are mainly from bregma − 6.84 mm to − 8.56 mm according to the rat brain atlas. Here, we collected sections from bregma − 7.20 to − 7.44 mm as the rostral PPN (rPPN), bregma − 7.68 to − 7.92 mm as the middle PPN (mPPN), and bregma − 8.16 to − 8.40 mm as the caudal PPN (cPPN). Based on the results of *in situ* hybridization and immunostaining, we counted ChAT, mCherry, vGluT2-mRNA, GAD2-mRNA, and CaMKIIα-mRNA cells. For each experimental group, cells of at least 6 sections from 3 animals were counted.

### Statistics

The graphics and numerical values of the averaged speed and normalized respiration rate are presented as the mean ± SEM. The graphics and numerical values of the percentage of cell number reported are presented as the mean ± SD. All experiments were performed in multiple batches of replication at least three times per experiment. *P <0.05, **P <0.01, and ***P <0.001, two-tailed paired *t-*test.

## Results

### PPN-vGluT2 Neurons are Recruited in Modulating Movement Patterns and Responses to Salient Environmental Stimuli

The PPN, located caudal to the SN and in proximity to the superior cerebellar peduncle, lies the midbrain-pons junction within the brainstem. Comprising glutamatergic (vGluT2), GABAergic (GAD2), and cholinergic (ChAT) neurons, the PPN exhibits a distinctive neurochemical topography [6–9]. Our simultaneous examination of these neuronal subtypes from the rostral to the caudal regions of the PPN revealed a uniform distribution of vGluT2 neurons throughout, while ChAT neurons predominated in the mid-to-caudal zones, and GAD2 neurons were chiefly found in the rostral and ventral segments (Fig. S1A-C). Notably, these populations were largely non-overlapping (Fig. S1B).

**Fig. 1:**
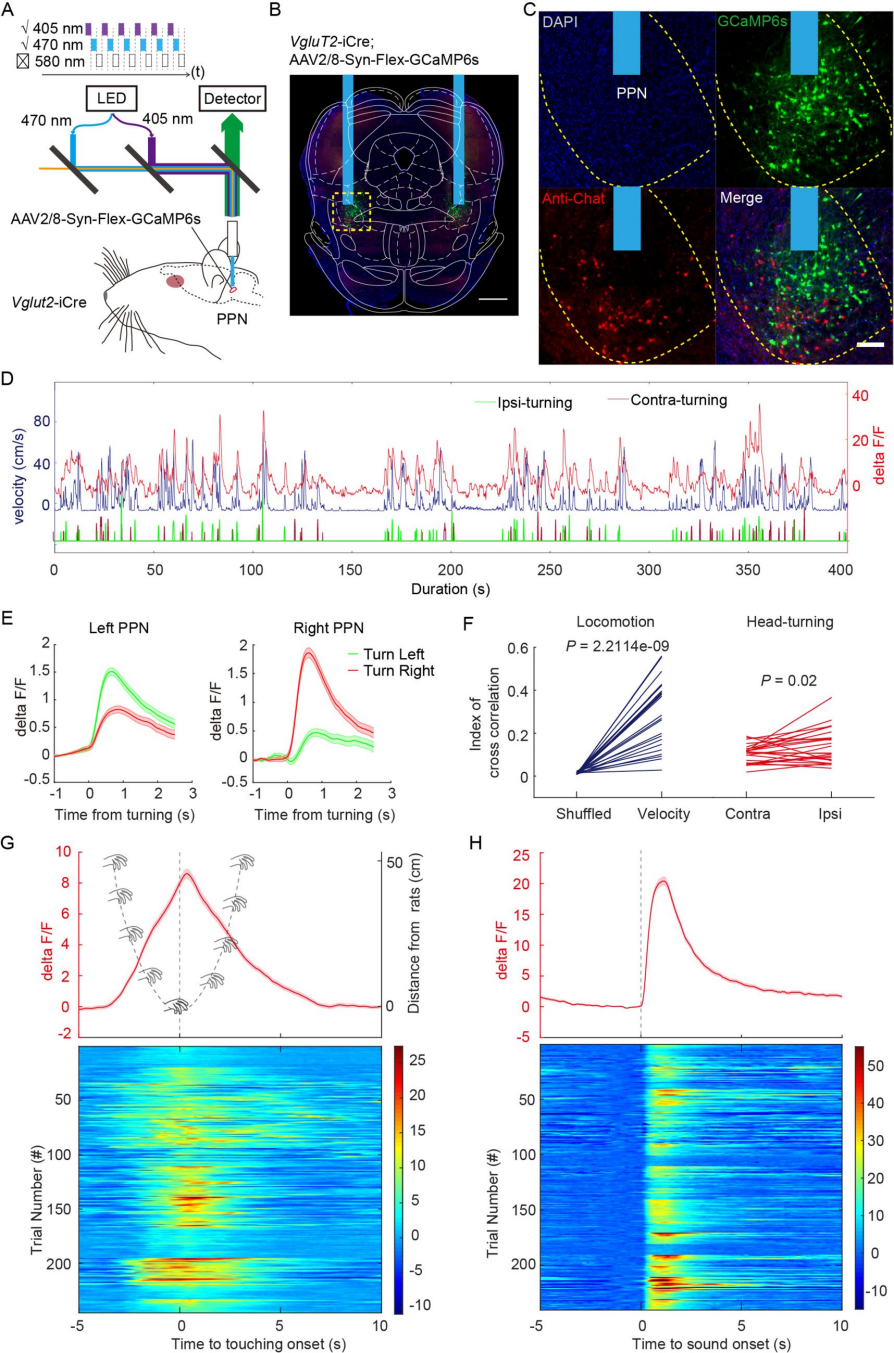
**Fiber photometry recording of rPPN-vGluT2 neuronal activity during locomotion and ipsilateral head-turning, and in response to salient stimuli. A** Schematic of fiber photometric recording of PPN vGluT2 neuronal activity. **B** Representative histology confirming the fiber placement and specificity of GCaMP6s expression in the PPN. Scale bar, 1000 μm. **C** Representative histology showing co-localization of GCaMP6s and ChAT in the PPN. Green, GCaMP6s; red, anti-ChAT. Scale bar, 200 μm. **D** Concurrent recording of locomotor velocity, directional head-turning, and PPN vGluT2 neuronal Ca^2+^ signals in freely-moving rats. Blue line, velocity of rat; red line, Ca^2+^ signals; green, ipsilateral head-turning; carmine, contralateral head-turning. **E** Averaged PPN-vGluT2 neuronal Ca^2+^ signal in response to head-turning behavior. Left panel, activity of the left PPN; right panel, activity of the right PPN, during left (red) and right (green) head-turning. Data are presented as the mean ± SEM. **F** Cross-correlation analysis between locomotor velocity and Ca^2+^ signals (left, *n = *24, paired *t-*test, *P = *2.2^−9^), and between head-turning and Ca^2+^ signals (right, *n = *24 paired *t-*test, *P = *0.02). **G** Activation of PPN-vGluT2 neurons during hand-touching stimulation. The red line is the averaged PPN-vGluT2 neuronal Ca^2+^ signal in response to hand-touching. Grey line, diagram of the distance of the hand from the rat during hand-touching, with the onset points as touching time points. Lower panel, heatmap of the PPN-vGluT2 neuronal Ca^2+^ signal in response to the onset of hand-touching. **H** Activation of PPN-vGluT2 neurons during salient sound stimulation. Red line, averaged PPN-vGluT2 neuronal Ca^2+^ signal in response to salient sound stimulation. Lower panel, heatmap of the PPN-vGluT2 neuronal Ca^2+^ signal in response to the onset of salient sound stimulation.

The prevailing literature presents inconclusive findings on whether PPN-vGluT2 neurons are implicated in the promotion or cessation of locomotion. It has been hypothesized that the activation of distinct PPN-vGluT2 subregions accounts for these mixed results [15, 20, 21]. To investigate this dichotomy, we obtained a *Vglut2*-iCre rat line and validated the specificity of iCre expression in PPN-vGluT2 neurons (Fig. S2). To elucidate the physiological role of rPPN-vGluT2 neurons in locomotion, we recorded *in vivo* Ca^2+^ signals using fiber photometry in freely-moving rats in an open field (Fig. 1A). After viral delivery of Cre-dependent GCaMP6s to the PPN rostrally, we confirmed that the localization of GCaMP6s was surrounded by ChAT-positive neurons, a marker for the PPN region (Fig. S1B, C). We also accurately verified the fiber implantation sites within the rostral and medial PPN across all subjects (Fig. S3). Analysis of the correlation between rPPN-vGluT2 neuronal activity and movement velocity revealed an increase in Ca^2+^ signals during locomotion, with a more pronounced rise during ipsilateral than contralateral turns (Fig. 1D-F). In addition, rPPN-vGluT2 neuronal activity increased when an object approached the rats, peaking (>8%) upon body contact (Fig. 1G). These neurons also responded to auditory stimuli (Fig. S4A, B), exhibiting a significant increase (~20%) in activity following a sudden sound (Fig. 1H, Movie S1, https://pan.cstcloud.cn/s/LcqY2YiQUU). Intriguingly, rPPN-vGluT2 neurons displayed an adaptive response to repeated acoustic stimulation, with reduced activity during subsequent exposures (Fig. S4C). Collectively, these findings demonstrate that rPPN-vGluT2 neurons play an integral role in locomotion and ipsilateral turning, and respond to salient environmental stimuli.

### Global Activation of rPPN-vGluT2 Neurons Arrests Movements

**Fig. 2:**
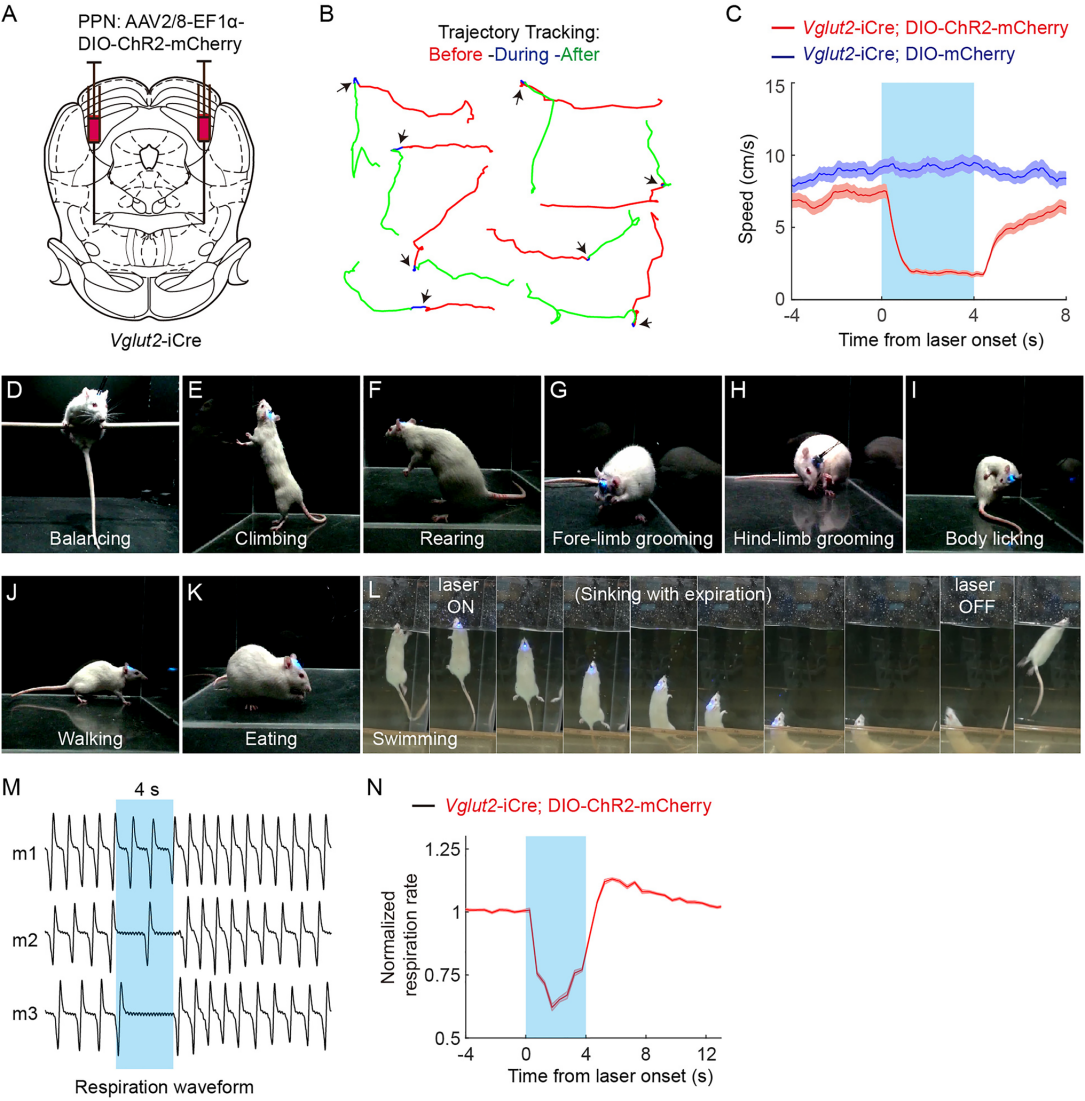
**Inhibition of voluntary and involuntary movements by activation of rPPN-vGluT2 neurons. A** Schematic of viral injection sites and fiber placement in the PPN. **B** Representative traces of 4-s locomotor trajectories before (red), during (blue), and after (green) optogenetic stimulation of PPN-vGluT2 neurons. **C** Averaged velocity shows that activation of PPN-vGluT2 neurons halts locomotion. Red line, experiment group with ChR2 expression, *n = *6. Blue line, control group with mCherry expression, *n = *6. Data are presented as the mean ± SEM. **D-L** Stationary postures are maintained during optogenetic activation of PPN-vGluT2 neurons. Rats are balancing (**D**), climbing (**E**), rearing (**F**), forelimb grooming (**G**), hindlimb grooming (**H**), body licking (**I**), walking (**J**), and eating (**K**) during optogenetic stimulation. Moreover, rats stop swimming and sink with a stationary posture when PPN-vGluT2 neurons are activated, and resume swimming after stimulation offset (**L**). **M, N** Example traces of involuntary respiration of anesthetized rats before, during, and after optogenetic activation of PPN-vGluT2 neurons (**M**) and normalized respiration rates (**N**) (*n = *9). Data are presented as the mean ± SEM.

Upon identifying the physiological role of rPPN-vGluT2 neurons, we endeavored to characterize their function through optogenetic manipulation. Initially, a Cre-dependent ChR2-expressing virus was injected into the rostral PPN of *Vglut2*-iCre rats (Fig. 2A). We confirmed the selective expression of ChR2 in rPPN-vGluT2 neurons (Fig. S5) and assessed the efficacy of activation using *in vivo* optrode recordings, *in vitro* brain slice recordings, and c-fos immunohistochemistry (Fig. S6). Subsequently, global optogenetic stimulation of rPPN-vGluT2 neurons in ambulatory rats resulted in the cessation of locomotion (Fig. 2B, C, Movie S2, https://pan.cstcloud.cn/s/LcqY2YiQUU). Importantly, this activation also interrupted other ongoing motor behaviors, including balancing, climbing, rearing, grooming, licking, walking, eating, and swimming (Fig. 2D-L, Movie S3, S4, S5, S6, https://pan.cstcloud.cn/s/LcqY2YiQUU). Conversely, inhibitory experiments with NpHR expression in rPPN-vGluT2 neurons showed no impact on locomotion (Fig. S7).

In addition, during rPPN-vGluT2 neuronal activation, we found that abdominal fluctuations in a freely-moving rat were markedly reduced or ceased (Movie S7, https://pan.cstcloud.cn/s/LcqY2YiQUU), suggesting the involvement of rPPN-vGluT2 neurons on respiratory movement control. To determine whether this respiratory suppression was independent of locomotor cessation, we measured respiratory rates in anesthetized rats and found a significant decrease or complete cessation upon stimulation of rPPN-vGluT2 neurons (Fig. 2M, N). Collectively, these findings indicate that rlPPN-vGluT2 neurons govern both voluntary and involuntary motor functions.

Notably, during optogenetic suppression of movement, rats adeptly maintained balance, even when suspended on a rod or climbing, without any forelimb contact with the ground (Fig. 2D, Movie S4, https://pan.cstcloud.cn/s/LcqY2YiQUU). Remarkably, they preserved these stationary postures for the duration of the stimulation, up to 20 s (Movie S5, https://pan.cstcloud.cn/s/LcqY2YiQUU). Furthermore, in an aquatic environment, rats ceased swimming and descended to the tank bottom, yet maintained their stationary posture throughout the stimulation (Fig. 2L, Movie S6, https://pan.cstcloud.cn/s/LcqY2YiQUU).

Post-stimulation observations revealed that motor functions resumed immediately after ceasing optogenetic activation, differing from fear-induced freezing behaviors [22, 23], which persist after the removal of the stimulus. Certain behaviors, like eating and swimming, recommenced immediately, whereas others, like grooming and rearing, did not (Movie S3, S4, S5, S6, S7, https://pan.cstcloud.cn/s/LcqY2YiQUU). These results underscore the role of rPPN-vGluT2 neurons in mediating not only the cessation of locomotion but also the modulation of a broader spectrum of voluntary and involuntary movements.

### The Dual Roles of rPPN-vGluT2 Neurons in Motor Modulation are Mediated Through Different Neural Circuits

**Fig. 3:**
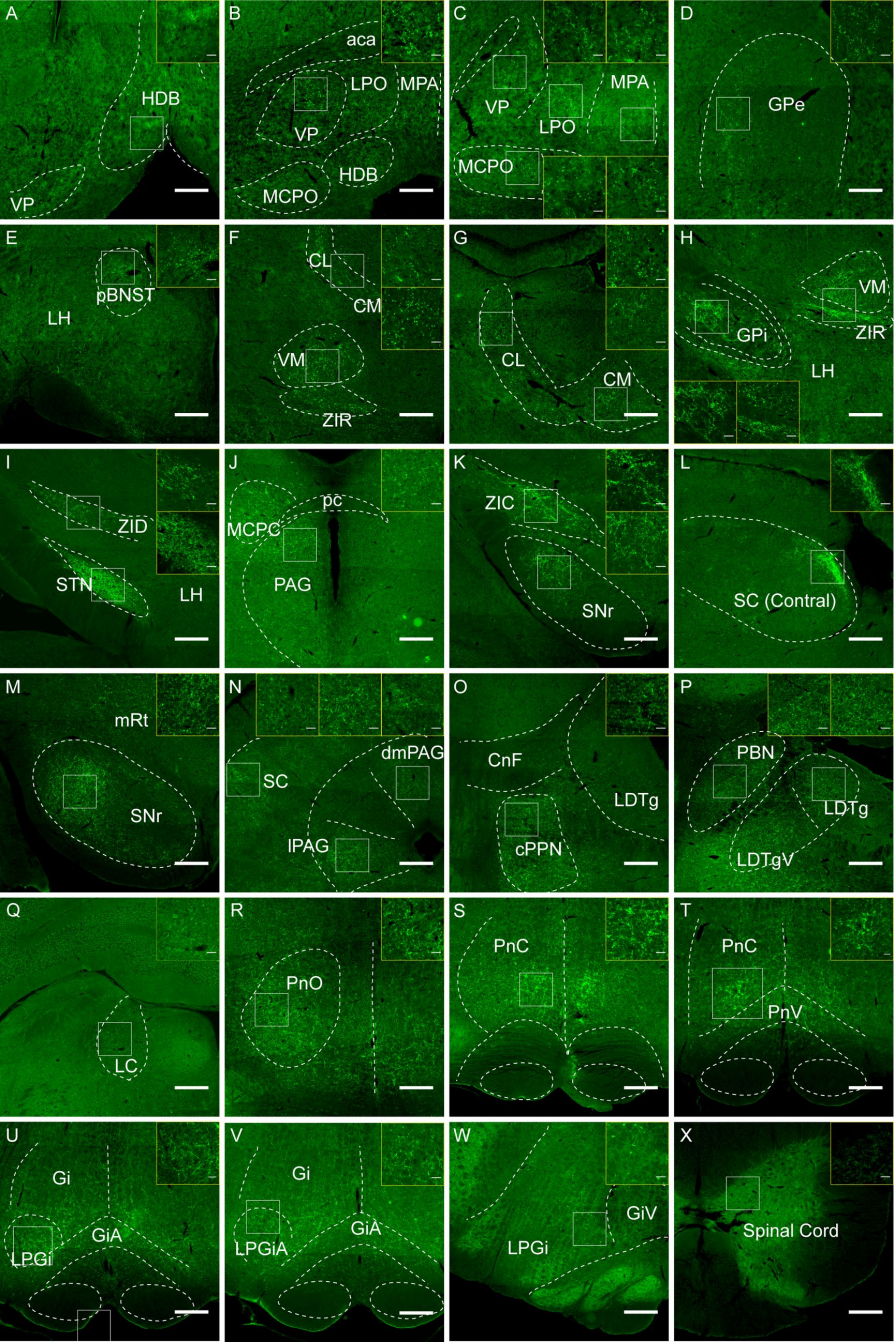
**Downstream targets of rPPN-vGluT2 neurons. A-X** Representative histology of downstream regions of PPN-vGluT2 neurons, including the horizontal limb of the diagonal band of Broca (HDB), ventral pallidum (VP), lateral preoptic area (LPO), medial preoptic area (MPA), magnocellular preoptic nucleus (MCPO), globus pallidus externus (GPe), posterior bed nucleus of stria terminalis (pBNST), centromedian nucleus (CM), centrolateral nucleus (CL), ventromedial nucleus (VM), ZI, rostral ZI (ZIR), dorsal ZI (ZID), caudal ZI (ZIC), globus pallidus internus (GPi), lateral hypothalamus (LH), subthalamic nucleus (STN), magnocellular nucleus of the posterior commissure (MCPC), superior colliculus (SC), mesencephalic reticular formation (mRt), periaqueductal gray (PAG), dorsomedial periaqueductal gray (dmPAG), lateral periaqueductal gray (lPAG), cuneiform nucleus (CnF), laterodorsal tegmental nucleus (LDT), parabrachial nucleus (PBN), locus coeruleus (LC), oral pontine reticular nucleus (PnO), PnC, ventral pontine reticular nucleus (PnV), GiA, Gi, lateral paragigantocellular nucleus (LPGi), gigantocellular reticular nucleus, ventral part (GiV), and the spinal cord. Scale bars, 1000 μm. The region of interest is windowed and enlarged in each panel. Scale bars, 60 μm.

Our initial findings in *Vglut2*-iCre/DIO-ChR2-mCherry rats, as evidenced by staining results, indicated that rPPN-vGluT2 neurons possess various efferent projections. To elucidate the precise synaptic terminals of these neurons, we injected a Cre-dependent viral vector encoding synaptophysin-EGFP, a synaptic terminal marker, into the rostral PPN of *Vglut2*-iCre rats (Fig. S8). Immunohistochemical analysis revealed that rPPN-vGluT2 neurons project predominantly to the horizontal limb of the diagonal band of Broca, ventral pallidum (VP), lateral preoptic area (LPO), medial preoptic area (MPA), magnocellular preoptic nucleus, globus pallidus externus (GPe), posterior bed nucleus of stria terminalis, centromedian nucleus, centrolateral nucleus, ventromedial nucleus, ZI, globus pallidus internus (GPi), lateral hypothalamus (LH), subthalamic nucleus (STN), magnocellular nucleus of posterior commissure, superior colliculus (SC), mesencephalic reticular formation, periaqueductal gray (PAG), cuneiform nucleus (CnF), laterodorsal tegmental nucleus (LDT), parabrachial nucleus (PBN), locus coeruleus (LC), oral pontine reticular nucleus, PnC, ventral pontine reticular nucleus, GiA, Gi, lateral paragigantocellular nucleus (LPGi), gigantocellular reticular nucleus, ventral part (GiV), and the spinal cord. (Fig. 3).

**Fig. 4:**
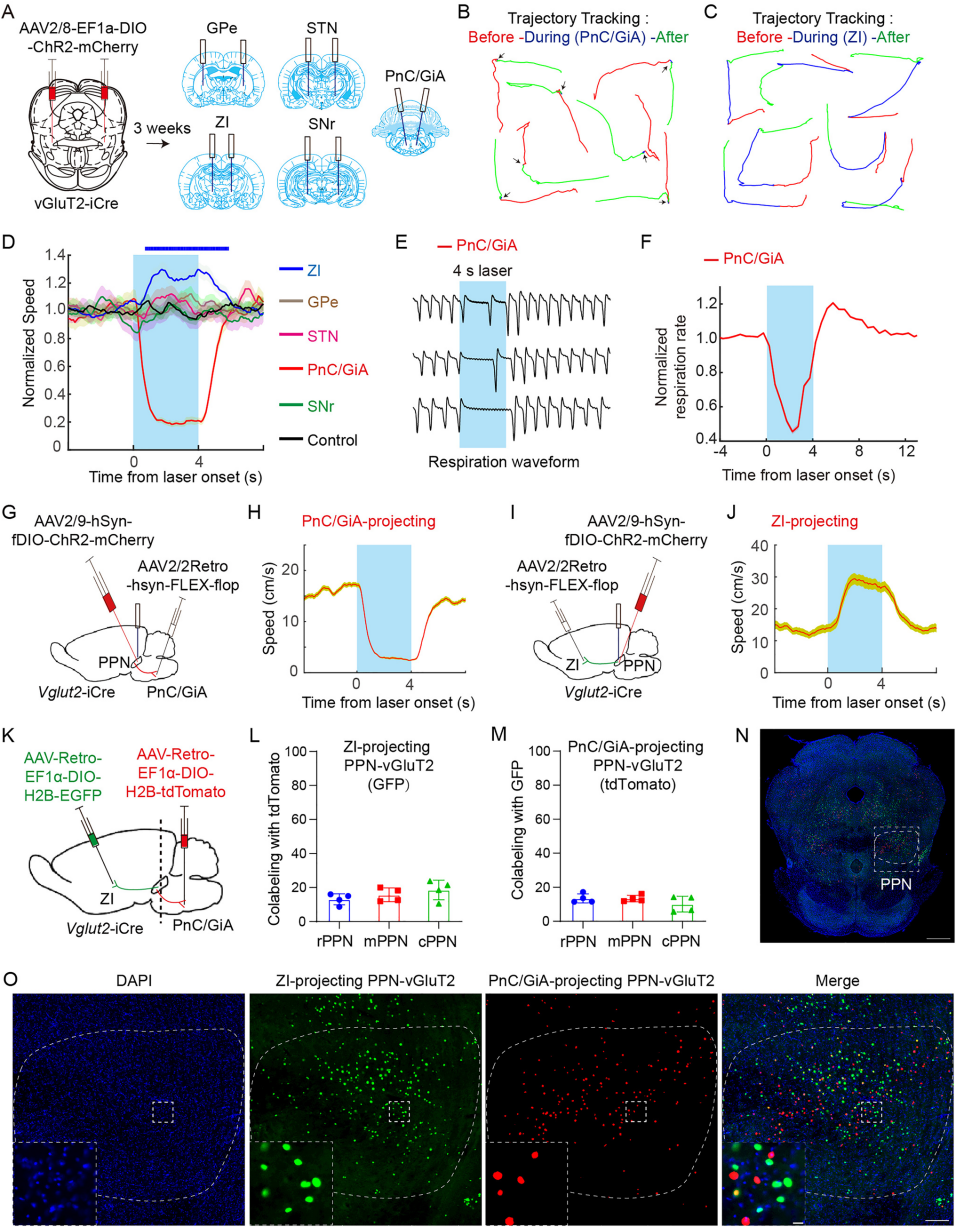
**The dual function of locomotor modulation is controlled by different neural circuits. A** Schematic of viral labeling (left) and fiber placement (right). **B, C** Representative traces of 4-s locomotor trajectories before (red), during (blue), and after (green) optogenetic stimulation of PPN-vGluT2 neuron terminals in the PnC/GiA (**B**) or ZI (**C**). **D** Changes in the averaged velocity resulting from optogenetic activation of PPN-vGluT2 neuron terminals in the PnC/GiA (red, *n = *7), GPe (brown, *n = *6), STN (pink, *n = *6), SNr (green, *n = *6), and ZI (blue, *n = *8). The control group (*n = *6) received laser stimulation to the ZI with mCherry-expression in PPN-vGluT2 neurons. Data are presented as the mean ± SEM. **E, F** Example traces of respiration of anesthetized rats before, during, and after optogenetic activation of PPN-vGluT2 neuron terminals in PnC/GiA (**E**) and normalized respiration rates (**F**) (*n = *7). Data are presented as the mean ± SEM. **G** Schematic of optogenetic activation of PnC/GiA-projecting PPN-vGluT2 neurons. **H** Changes in averaged velocity during locomotion induced by optogenetic activation of PnC/GiA-projecting PPN-vGluT2 neurons (*n = *6). Data are presented as the mean ± SEM. **I** Schematic of optogenetic activation of ZI-projecting PPN-vGluT2 neurons. **J** Changes in averaged velocity during locomotion induced by optogenetic activation of ZI-projecting PPN-vGluT2 neurons (*n = *4). Data are presented as the mean ± SEM. **K** Schematic of labeling neuronal nuclei of PnC/GiA-projecting and ZI-projecting PPN-vGluT2 neurons. **L, M** Quantitation of the overlapped neurons among PnC/GiA-projecting and ZI-projecting PPN-vGluT2 neurons. **L**, the percentage of ZI-projecting PPN-vGluT2 neurons (GFP) co-labeled with PnC/GiA-projecting PPN-vGluT2 neurons (tdTomato) in the rPPN, mPPN, and cPPN; **M**, the percentage of PnC/GiA-projecting PPN-vGluT2 neurons (tdTomato) co-labeled with ZI-projecting PPN-vGluT2 neurons (GFP) in the rPPN, mPPN, and cPPN. **N, O** Representative histology showing the different distribution of PnC/GiA-projecting (red, tdTomato) and ZI-projecting (green, GFP) PPN-vGluT2 neurons. **N**, Representative histology in the rPPN. Scale bar, 1000 μm; **O**, Window from **N**. Scale bar, 200 μm. The left*-*lower panel, enlarged window. Scale bar, 20 μm.

To identify which descending pathways are implicated in the cessation of locomotion and other types of movement, we administered a virus carrying Cre-dependent ChR2-mCherry bilaterally into the rostral PPN of *Vglut2*-iCre rats. We subsequently implanted optical fibers above the projection sites of rPPN-vGluT2 neurons in downstream regions. Optogenetic stimulation of these terminals within the GPe, STN, SNr, PnC/GiA, and ZI was performed (Fig. 4A). Our results indicated that termination of locomotion occurred exclusively upon optogenetic activation of the terminals in the PnC/GiA, with no effect from other regions. Conversely, locomotion promotion was facilitated by stimulation of the terminals in the ZI (Fig. 4B-D). In addition, a reduction in respiratory rate was found during activation of the projections in the PnC/GiA. Notably, behaviors such as walking, grooming, and rearing, which were discontinued during photoactivation, promptly resumed pos*t-*activation (Movie S8, https://pan.cstcloud.cn/s/LcqY2YiQUU).

To further substantiate the role of the projections of rPPN-vGluT2 neurons to PnC/GiA and ZI pathways, we injected a retrograde virus expressing Cre-dependent flp into these two regions and delivered a virus containing flp-dependent ChR2-mCherry into the rostral PPN (Fig. 4G, I). Activation of the PnC/GiA-projecting PPN-vGluT2 neurons halted movement, whereas stimulation of the ZI-projecting neurons enhanced locomotion (Fig. 4H, J; Movie S9, https://pan.cstcloud.cn/s/LcqY2YiQUU). To elucidate the distinct contributions of these circuits to motor modulation, we traced the soma distribution of the two neuronal subpopulations within the PPN. We administered viruses carrying Cre-dependent fluorescent proteins (nuclear-located EGFP for ZI and nuclear-located tdTomato for PnC/GiA) to precisely target and visualize the locations of PPN-vGluT2 neurons (Fig. 4K). We determined that the ZI-projecting neurons were predominantly distinct from those projecting to the PnC/GiA in the PPN (Fig. 4L-O).

In summary, our findings show that the PnC/GiA is the key downstream target mediating the rPPN-vGluT2 neurons’ ability to arrest locomotion and general motor activity. Conversely, the rPPN-vGluT2 projections to the ZI facilitate locomotion. These results highlight the dual role of rPPN-vGluT2 neurons in motor modulation, which is mediated by discrete neural circuits.

### PPN-vGluT2 Neurons Modulate the ‘Pause-Switch-Play’ Motor Pattern

With the global activation of rPPN-vGluT2 neurons or their terminals in the PnC/GiA, we have shown that arrest behaviors can be induced. However, these behaviors resulted from activating the two targets displayed differently. Global activation of the neurons themselves primarily leads to a transient cessation of motor activity, followed by a resumption with a potential motor pattern change in behavior after the cessation of optogenetic stimulation (Movie S3, S4, S5, S6, https://pan.cstcloud.cn/s/LcqY2YiQUU). In contrast, activation of the terminals in the PnC/GiA mainly results in a continuation of the preceding action once stimulation is offset (Movie S8, https://pan.cstcloud.cn/s/LcqY2YiQUU).

**Fig. 5:**
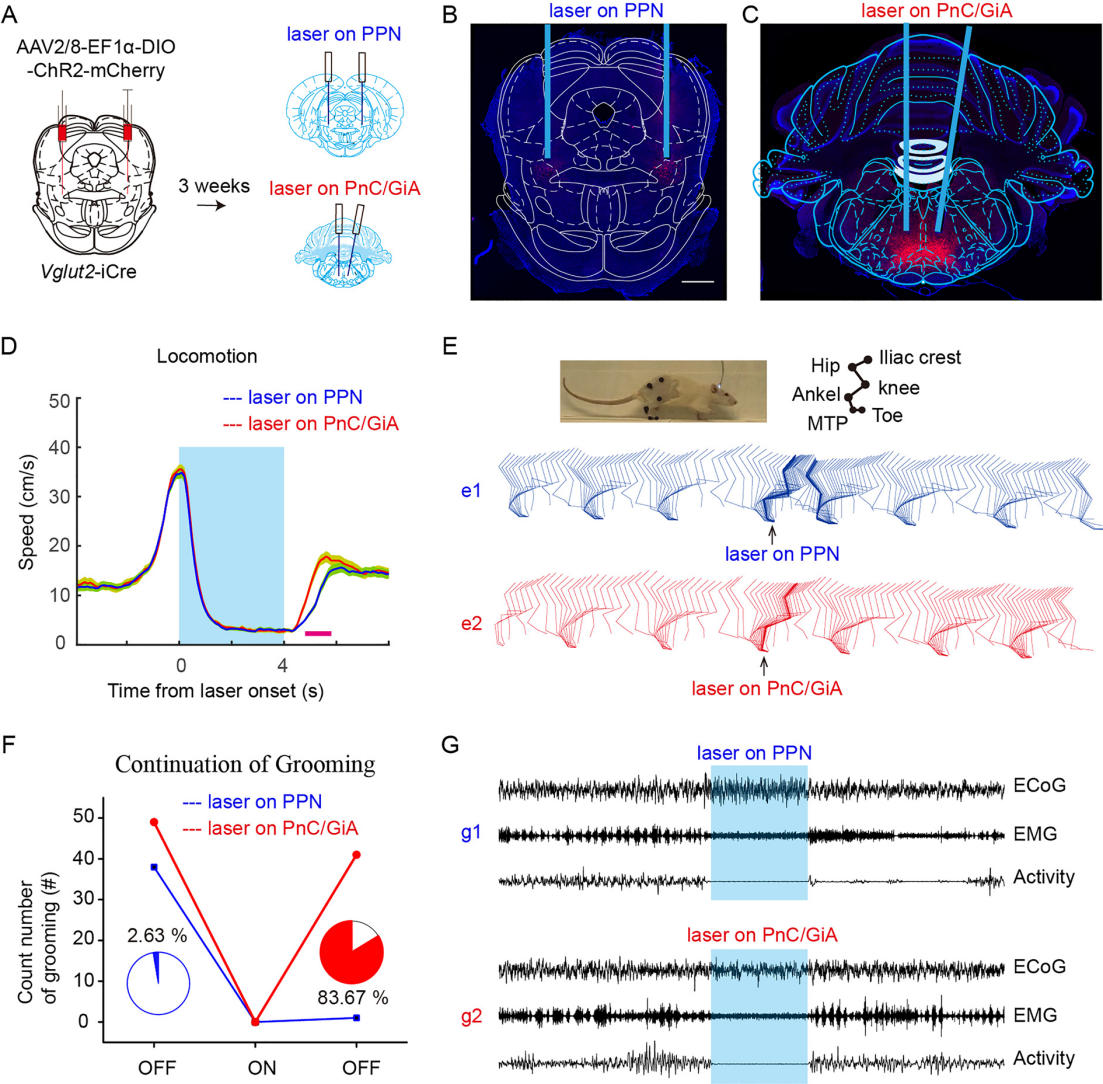
**PPN-vGluT2 neurons projecting to the PnC/GiA are responsible for pause-and-play pattern arrest behavior. A** Schematic of virus injection and fiber placement. **B** Representative histology of a coronal section showing ChR2-mCherry expression (red) in somata of PPN-vGluT2 neurons. Blue rectangle, fiber position. Scale bar, 1000 μm. **C** Representative histology of a coronal section showing PPN-vGluT2 neuron terminals in the PnC/GiA with ChR2-mCherry expression (red). Blue rectangle, fiber position. **D** Changes in the averaged velocity resulting from optogenetic activation of PPN-vGluT2 neuron somata (blue line) or their terminals in the PnC/GiA (red line) (*n = *7). Data are presented as the mean ± SEM. Pink bar, time window with a significant change at the *P*<0.05 level. **E** Changes in the pause-and-play pattern of hindlimb dynamics resulting from optogenetic activation of PPN-vGluT2 neuron somata (blue line) or their dendrites in the PnC/GiA (red line) during locomotion in a linear corridor. The arrow indicates the period of pause. **F** Changes in the pause-and-play pattern of grooming resulting from optogenetic activation of PPN-vGluT2 neuron somata (blue line, 1/38) or their dendrites in the PnC/GiA (red line, 41/49). **G** Changes in representative ECoG, EMG, and activity signals of ongoing grooming resulting from optogenetic activation of PPN-vGluT2 neuron somata (blue line) or their dendrites in the PnC/GiA (red line).

To further elucidate the role of rPPN-vGluT2 neurons and their projections to the PnC/GiA, a comparative investigation was conducted. We administered a virus enabling Cre-dependent ChR2-mCherry expression into the bilateral rPPN, and implanted optical fibers targeting both the bilateral PPN and PnC/GiA in *Vglut2*-iCre rats (Fig. 5A-C). To assess the differential effects on locomotor modulation, rats were trained to run in a 1.6-meter-long and 0.6-meter-wide open field for water rewards, with optogenetic stimuli applied at the midpoint of their run, randomly targeting either the PPN or PnC/GiA. Our findings showed that rats ceased their running activity under both experimental conditions (Fig. 5D). However, they resumed running faster after stimulation offset in the PnC/GiA compared to the PPN (Fig. 5D). In addition, we examined the hindlimb dynamics under both conditions by training and recording the rats as they ran through a transparent linear corridor. The results indicated a temporary halt after stimulation offset in the PPN, whereas in the PnC/GiA, rats continued their movement smoothly (Fig. 5E). Grooming behavior was also analyzed, revealing that rats tended to persist with grooming in most cases (83.67%) after stimulation offset in the PnC/GiA, unlike in the PPN condition (2.67%) (Fig. 5F). Furthermore, electromyographic analysis of the neck muscles showed a switch from clustered activity during grooming before stimulation to a non-clustered pattern following stimulation offset in the PPN, while in the PnC/GiA, the clustered activity persisted both before and after stimulation (Fig. 5G).

Taken together, our research demonstrates that rPPN-vGluT2 neuronal activation can induce a ‘pause-switch-play’ pattern of motor modulation—halting movement during stimulation and resuming with different actions thereafter. In addition, we have shown that activation of the terminals in the PnC/GiA does lead to a ‘pause-and-play’ pattern of motor modulation—stopping movement during stimulation and continuing the same action once stimulation is offset.

### Dissecting the Inputs of rPPN-vGluT2 Neurons

**Fig. 6:**
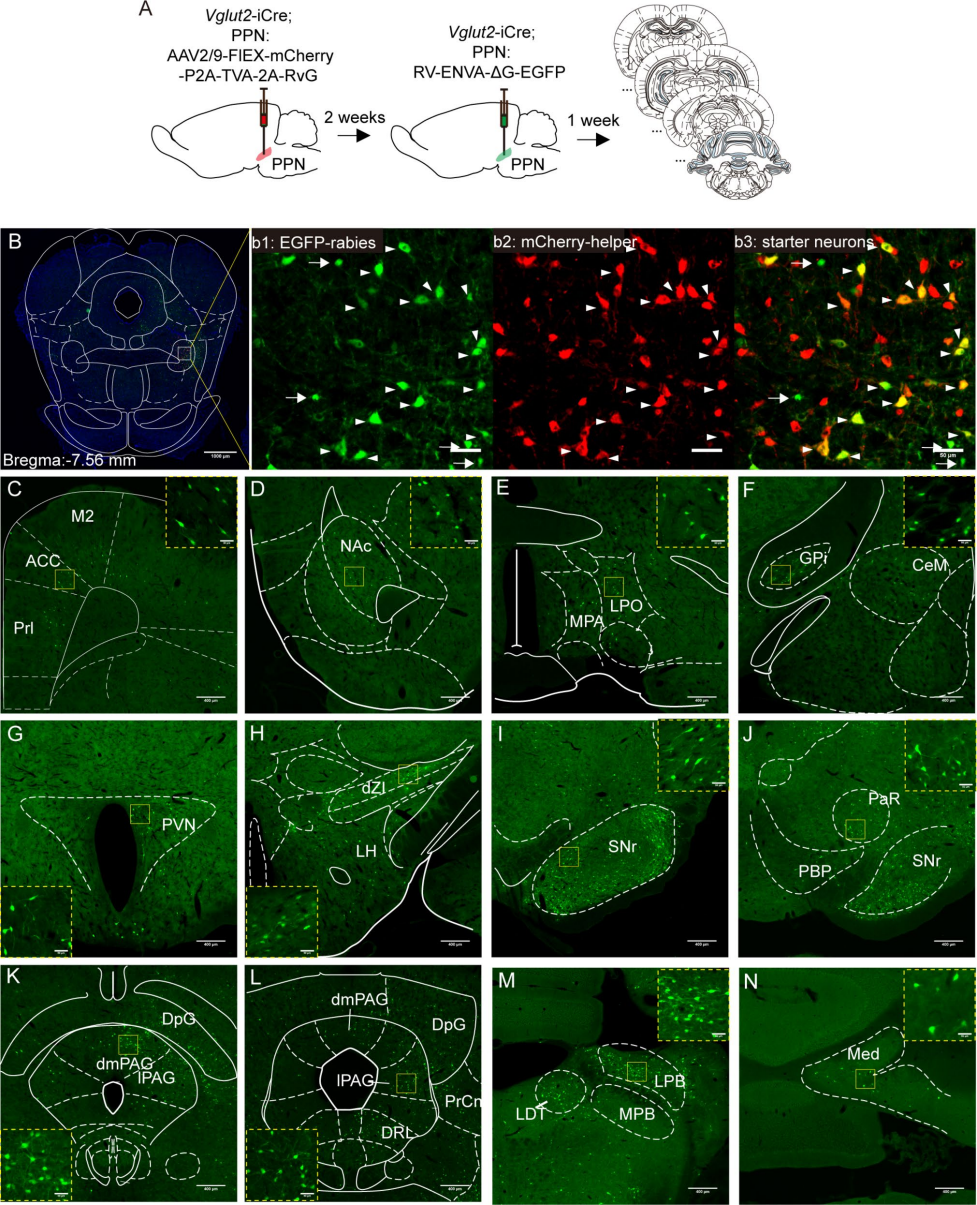
**Inputs of rPPN-vGluT2 neurons. A** Schematic of injections of helper and rabies viruses into the PPN. **B** Representative histology showing the viral injection site. B1, green is a signal of rabies labeling; b2, PPN-vGluT2 neurons with helper virus labeling; b3, yellow cells are the start neurons for retrograde trans-synaptic tracing. **C-N** Representative histology of areas upstream of PPN-vGluT2 neurons, including secondary motor cortex (M2), anterior cingulate cortex (ACC), prelimbic cortex (Prl), nucleus accumbens (NAc), MPA, LPO, GPi, central medial amygdala (CeM), paraventricular nucleus (PVN), LH, ZI, SNr, pararubral nucleus (PaR), SC, PAG, dorsal raphe nucleus (DRN), lateral parabrachial nucleus (LPB), medial parabrachial nucleus (MPB), LDT, and medial cerebellum (Med).

In the preceding sections, we have shown that PPN-vGluT2 neurons are involved in multiple functions through various neural circuits. To further elucidate the intricacies of the connections, we proceeded to identify the specific inputs to rPPN-vGluT2 neurons. Initially, we applied monosynaptic retrograde tracing to these neurons (Fig. 6A). The starter neurons, labeled with a helper virus and the rabies-ΔG virus, were precisely confined to the rostral PPN region (Fig. 6B). Monosynaptic inputs to the PPN were traced to both proximal sources within the PPN itself (Fig. 6B) and long-range projections to the PPN. The latter encompassed but were not limited to, the prelimbic cortex (Prl), anterior cingulate cortex, secondary motor cortex, nucleus accumbens, VP, LPO, GPi, paraventricular nucleus, ZI, SNr, dmPAG, lPAG, PrCnF, lateral dorsal raphe nucleus, PBN, and medial cerebellar nucleus (Fig. 6C-N). Notably, inputs originating from the SNr and PAG were the most numerous.

For a more targeted analysis of the inputs to PnC/GiA-projecting PPN-vGluT2 neurons, we injected a retrograde Cre-dependent helper virus into the PnC/GiA and the rabies-ΔG virus directly into the PPN of *Vglut2*-iCre rats (Fig. S9A, B). The upstream inputs for these specific PPN-vGluT2 neurons largely mirrored those identified in the broader PPN-vGluT2 neuron population (Fig. S9D-R), with the additional discovery of inputs from the cerebellar cortex (Fig. S10). Interestingly, we discerned that the PPN-projecting PAG neurons differed from the PnC/GiA-projecting PAG neurons in their location; the former predominated within the dmPAG and lPAG, whereas the latter were mainly found in the ventrolateral periaqueductal grey (vlPAG) (Fig. S9R). Collectively, these findings confirm that PPN-vGluT2 neurons are integrated into a complex network of inputs from the frontal cortex, basal ganglia, midbrain, brainstem, and cerebellum.

**Fig. 7:**
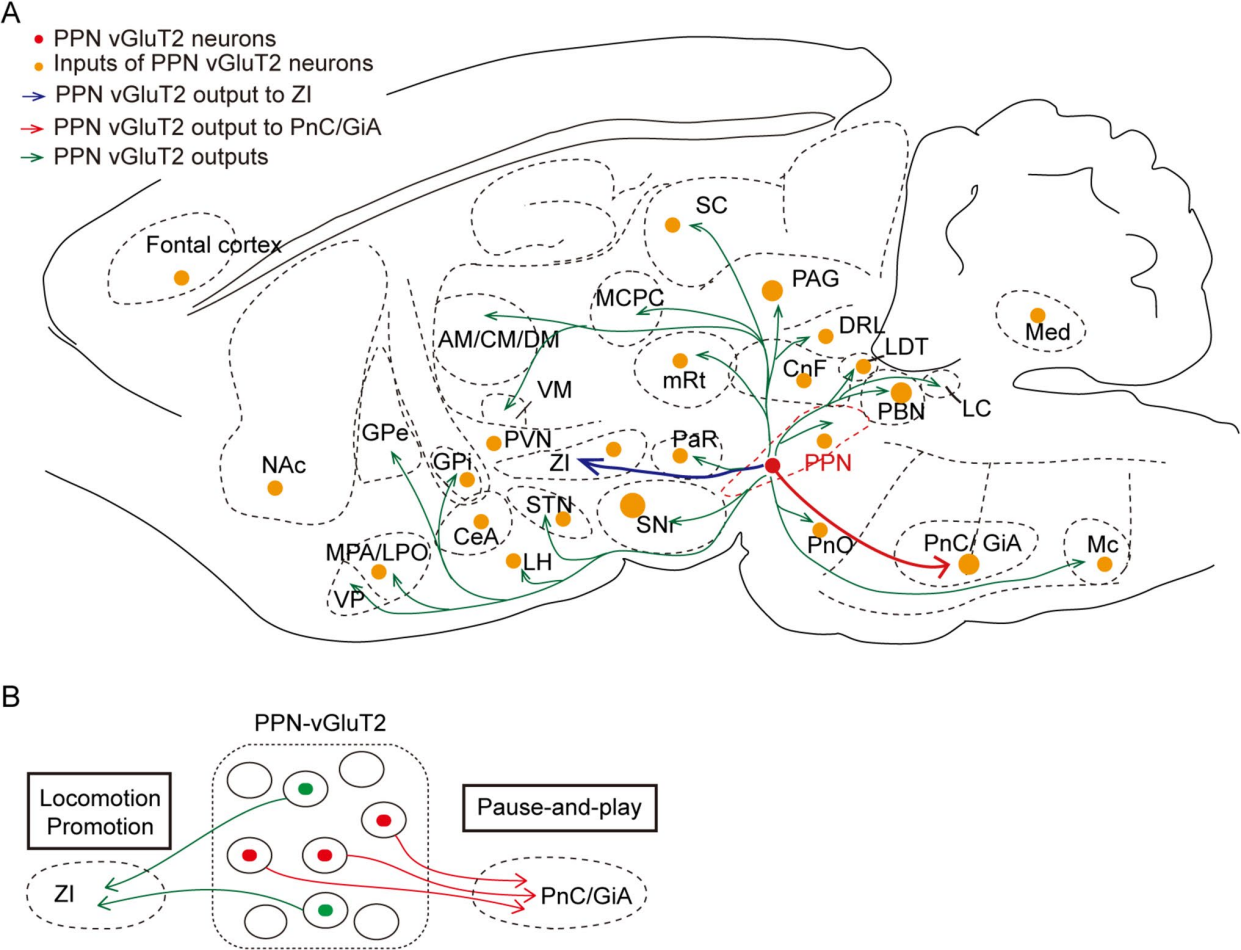
**Supraspinal circuits via rPPN glutamatergic neurons modulate multiple motor actions. A** Connectome of PPN-vGluT2 neurons in the whole brain. Arrows represent the downstream outputs of PPN vGluT2 neurons; yellow dots represent the upstream inputs. **B** Schematic of different motor modulation underlying different circuits of PPN-vGluT2 neurons. The projection from PPN-vGluT2 to ZI promotes locomotion, and the projection to PnC/GiA plays an important role in pause-and-play pattern arrest behavior.

In summary, our investigations into the connectome of PPN-vGluT2 neurons have revealed diverse supraspinal circuits that modulate motor functions through glutamatergic signaling within the rostral PPN (Fig. 7).

## Discussion

The present study has uncovered that various supraspinal circuits, mediated by rPPN-vGluT2 neurons, contribute to diverse aspects of motor modulation. These neurons are recruited in coordinating locomotion, ipsilateral turning, and responses to salient stimuli. Specifically, activation of rPPN-vGluT2 projections to the ZI facilitates locomotion, whereas their projections to the PnC/GiA result in the ‘pause-and-play’ pattern of motor arrest. Notably, the global activation of rPPN-vGluT2 neurons themselves is involved in modulating the ‘pause-switch-play’ motor pattern.

### Functional Diversity of PPN-vGluT2 Neurons in Motor Modulation

The PPN, located in the rostral ventral MLR, is recognized as a pivotal relay nucleus connecting the basal ganglia and the spinal cord in motor control. Electrophysiological studies applying micro-stimulation have demonstrated that the PPN facilitates locomotion across various species, including cats, rats, and monkeys [3]. Yet, these findings are not unanimous, some studies report contradictory effects—either inhibition [24, 25] or a dual influence [26] on locomotion. Such inconsistencies are believed to be attributable to variations in stimulus parameters such as frequency, intensity, duration, and precise PPN targeting [20, 21].

Over the past decade, a heightened focus on the cellular specificity within the PPN and MLR has yielded new insights into their roles in motor modulation. Comprising vGluT2, ChAT, and GABAergic neurons, the PPN displayed a heterogeneous distribution of these neuronal types (Fig. S1), corroborating earlier research [6–9]. It has been increasingly recognized that distinct neuron subtypes within the PPN differentially influence locomotion [10, 11]. However, the specific contribution of PPN-vGluT2 neurons remains elusive. Studies in mice have shown that PPN-vGluT2 neurons can either enhance [11, 12] or inhibit locomotion [13, 14], or even exert a combined effect [15]. Notably, recent reports have shown that activation of MLR-Rbp4^+^ or PPN-Chx10^+^ neurons—both main subtypes of vGluT2—can arrest movement in mice [16, 17]. This variability in outcomes likely stems from the experimental manipulation of distinct PPN subdomains, each associated with particular neuron subtypes and their projections.

Our findings (Figs 6, S9, S10), along with previous literature [10, 11, 14, 17, 18], show that PPN-vGluT2 neurons receive extensive inputs from the frontal cortex, thalamus, hypothalamus, basal ganglia, midbrain, and cerebellum, and project widely to various brain regions, including the basal forebrain, thalamus, basal ganglia, midbrain, brainstem, and spinal cord. Recently, Ferreira-Pinto *et al.* have further reported that SN-projecting MLR Rbp4^+^ neurons, which are primarily vGluT2-positive, stop locomotion upon activation. Conversely, activating MLR-vGluT2 neurons projecting to the spinal cord induces body extension in mice, and those projecting to the medulla enhance locomotion [16]. Our results revealed that activating the entire population of rPPN-vGluT2 neurons inhibits locomotion, a response similar to that found when stimulating PnC/GiA-projecting PPN-vGluT2 neurons, yet distinct from that of ZI-projecting neurons, which instead facilitate locomotion (Figs 2, 4G-L). These findings collectively support the presence of anatomically and functionally distinct vGluT2 neuron subpopulations within the PPN and MLR. To elucidate the multiple functions of the PPN in motor modulation, pursuing cell-type-specific research represents a critical and promising avenue.

### The PPN-vGluT2-to-PnC/Gi Projection is Responsible for the Arrest of the ‘pause-and-play’ Motor Pattern

A recent investigation by Goni-Erro *et al.* has identified Chx10^+^ neurons, which also project to multiple downstream targets, as mediators of a global motor arrest behavior [17]. This behavior, termed ‘pause-and-play’ pattern arrest behavior, is notable for its rapid onset and offset [17]. Our current study reveals that the stimulation of PPN-vGluT2 neurons projecting to the PnC/GiA elicits a cessation of movement, preserving existing postures during the stimulation period and permitting the resumption of prior activity upon the stimulus offset (Fig. 5 and Movie S8, https://pan.cstcloud.cn/s/LcqY2YiQUU). This response mirrors the previously described ‘pause-and-play’ pattern. In addition, we found that this arrest behavior coincides with the suspension of involuntary respiratory movements (Figs 2M, N, and 4E, F), paralleling the effects of apnea in PPN Chx10^+^ neurons [17].

It is important to distinguish the ‘pause-and-play’ pattern from the freezing behavior induced by fear, such as that triggered by activating glutamatergic neurons in the SC or their projections to the lateral posterior nucleus of the thalamus, which results in prolonged immobility after stimulation [22, 23]. Moreover, the ‘pause-and-play’ pattern differs from arrest behaviors associated with resting postures or collapse, such as those induced by activating GlyT2 neurons in the Gi/GiV [27]. In mice, arrest behaviors can also be induced by the activation of vGluT2 neurons in the vlPAG, characterized by a cessation of movement during stimulation with subsequent resumption pos*t-*stimulation [28]. Goni-Erro *et al*. further demonstrated that the arrest behavior elicited by vlPAG Chx10^+^ neurons differs from that induced by PPN Chx10^+^ neurons; the former involves a longer latency and a slower re-initiation of locomotion, coupled with an initial rapid heart rate upon stimulus onset [17]. In our study, we found that activation of the projection from rPPN-vGluT2 neurons to the PnC/GiA did not switch the motor pattern during or after stimulation (Fig. 5). Furthermore, our results revealed that rats did not produce 22-kHz ultrasonic vocalizations (data not shown), which indicate negative emotions [29], in response to optogenetic stimulation. Therefore, we propose that the ‘pause-and-play’ pattern arrest behavior represents a form of motor modulation mediated by the descending projection from rPPN-vGluT2 neurons to the PnC/GiA. This process occurs independently of emotional interference.

Meanwhile, upstream of PnC/GiA-projecting PPN-vGluT2 neurons are the dmPAG and lPAG (Figs 6, S9), and upstream of PAG-vGluT2 neurons of the PnC/GiA are the lPAG and vlPAG (Fig. S9). It’s notable that these two group neurons are different subpopulations of PAG neurons (Fig. S9R). Functionally, the direct pathway from PAG to the magnocellular nucleus of the medulla is responsible for freezing behavior [28], while the indirect pathway from PAG-PPN-PnC/GiA is responsible for pause-and-play arrest behavior.

The descending output of PPN-vGluT2 neurons to the PnC/GiA encompasses the PnC, GiA, Gi, and LPGi in the ventral brainstem. Evidence suggests that activation of LPGi-vGAT or LPGi/GiA-GlyT2 neurons can arrest locomotion while maintaining muscle tone [27]. In addition, activation of V2a-specific excitatory neurons in the rostral Gi has been shown to terminate locomotion in mice [30]. Usseglio *et al*. discovered that GiA V2a neurons with projections to the lumbar, but not cervical, spinal cord can specifically halt locomotion [31]. Analogously, in lampreys, a comparable ‘stop cell’ has been identified within the middle rhombencephalic reticular nucleus, which is functionally similar to V2a neurons in the Gi, acting to suppress locomotion [32] and being regulated by vGluT2 MLR neurons [33]. Consequently, to elucidate the neural mechanisms underlying the ‘pause-and-play’ pattern arrest behavior, further research may be required to ascertain whether LPGi-vGAT, LPGi/GiA-GlyT2 or GiA-V2a neurons serve as downstream targets of PPN-vGluT2 neurons.

### The Elusive Causality Between PPN-vGluT2 Neurons and Arrest Behavior

Establishing a complete causal link needs examining physiological functions through both gain-of-function and loss-of-function studies. The PPN or MLR vGluT2 neurons have been reported to be implicated in the facilitation of movement. Roseberry *et al.* reported that MLR vGluT2 neurons initiate and maintain locomotion through neural firing, which encompassed encoding, activation, and inhibition experiments [10]. Complementarily, Caggiano and colleagues provided evidence that the CnF and PPN vGluT2 neurons are involved in high-speed running and low-speed exploratory behaviors, respectively, with findings substantiated by causal data [11].

Despite this, recent studies have suggested that PPN-vGluT2 neurons play a role in terminating locomotion [13–17], yet no single study has definitively established a causal link between PPN-vGluT2 neuronal activity and the cessation of movement. It has been reported, through single-unit recordings, that vGluT2 neurons in the MLR or PPN can encode a stationary state [10, 11]. However, it is important to differentiate between the stationary state (where the speed is zero) and the locomotor termination caused by the PPN or MLR; the former appears to be an automatic process inherent to locomotion, while the latter is associated with goal-directed voluntary movement. In a study of MLR Rbp4 neurons, Ferreira-Pinto *et al*. found that inhibiting these neurons leads to uncoordinated body movements, and activating them results in the cessation of ongoing movement, with predominant recruitment during forelimb behaviors such as handling and grooming [16]. This physiological basis does not align with the reported effects of activation-evoked stopping, casting doubt on the conclusion that MLR-induced movement suspension is convincing. More recently, Goni-Erro *et al*. demonstrated that PPN Chx10^+^ neurons, a subset of vGluT2 neurons, mediate a ‘pause-and-play’ pattern indicative of global motor arrest [17]. While they did not establish direct causation between the activity of PPN Chx10^+^ neurons and arrest behavior, their findings suggest that arrest behavior is a rapid physiological response to salient environmental cues [17]. Our research contributes evidence that rPPN-vGluT2 neurons are significantly recruited in response to such cues, specifically to sudden auditory stimuli, as indicated by a ~20% change in Ca^2+^ signaling (Figs 1G, H, and S4C). However, our study is limited by the inability to discern the rapid reaction period in response to salient cues from other processes. One possible explanation is that being still after salient stimuli may offer an important opportunity to assess the environmental conditions. Tseng and colleagues have concluded that the freezing state during the risk assessment period causes not only an immediate stilling of all movement but also increased muscle tone and behavioral vigilance to the threat, with reduced breathing and heart rates [34]. This is similar to the performance induced by activation of rPPN-vGluT2 neurons in our study (Fig. 2M, N). Moreover, it has been reported that mice perform risk-assessment behavior with a stretch-attend posture as they stay around the potential threat, and this process involves the dmPAG [35]. In our study, rPPN-vGluT2 neurons received a projection from dmPAG, which may provide anatomical support for the underlying neural circuits.

Another gap in causal understanding exists between PPN-vGluT2 neuronal activity and freezing of gait (FOG) in PD. The PPN has been targeted by DBS to manage FOG in advanced PD [5], yet the precise nature of PPN activity or abnormal firing patterns during PD progression remains unknown. The STN and GPi are recognized as effective DBS targets for enhancing motor function in PD, informed by knowledge of their overactivity in patients [36]. Although the mechanisms underlying DBS remain under study, the prevailing hypothesis is that high-frequency DBS suppresses hyperactivity and disrupts abnormal basal ganglia circuit synchronization, thereby restoring function [37–39]. Studies on rodent models of PD, induced by 6-hydroxydopamine (6-OHDA), have not clarified the pathophysiology of the PPN [40]. Studies have shown that glutamatergic PPN neurons are hyperactive following 6-OHDA lesions [41] and exhibit phase-locking with beta oscillations [42]—a pathological hallmark of PD [36]. This suggests that PPN-DBS could mitigate FOG by reducing PPN hyperactivity in PD patients, but the underlying mechanisms warrant further investigation.

### Implications of PPN-DBS in PD Patients

The PPN has been applied as a potential target for DBS in PD patients unresponsive to dopaminergic medication and STN/GPi-DBS [43]. Initial trials demonstrated that PPN-DBS improved motor ability in patients with advanced PD [44–46]. While subsequent double-blind trials confirmed PPN-DBS’s efficacy in enhancing gait and balance to some extent, they did not conclusively show improvements in ambulation [47, 48]. Notwithstanding very high individual variability, an extensive compilation of clinical case studies suggests that PPN-DBS enhances balance [49, 50]. Our current research reveals that activating rostral and middle PPN-vGluT2 neurons induces a halting behavior characterized by global motor arrest (Fig. 2, Movie S2, S3, S4, S5, S6, https://pan.cstcloud.cn/s/LcqY2YiQUU), where rats are rendered immobile despite external stimuli. These findings lead us to hypothesize that the activation of PPN-vGluT2 neurons may mirror the pathophysiological state of the PPN in PD patients, positioning it as a viable target for DBS to alleviate FOG by reducing the hyperactivity of excitatory PPN neurons.

Motor commands originating in the basal ganglia are relayed to the SNr and are subsequently transmitted to the medulla through the dorsal MLR, encompassing structures such as the CnF and dorsal caudal PPN [10, 12, 51]. This implies that STN/GPi-DBS might depend on the integrity of the neural circuits extending from the SNr through the dorsal MLR to the medulla. Recently, evidence has supported the hypothesis that the CnF can be a target for DBS to promote locomotion [52]. Thus, simultaneous DBS of the STN/GPi and PPN may offer a synergistic approach at the neural circuit level, with the former targeting the SNr-CnF-medulla circuits to promote movement and the latter focusing on the PPN-PnC/GiA to alleviate FOG. These insights establish a foundation for innovative DBS treatment protocols, which may include personalized stimulation of specific PPN subregions in conjunction with the STN/GPi to improve locomotion, gait, and balance.

In addition, it has been noted that patients with PD experiencing gait freezing are unable to walk while engaged in conversation [53]. Moreover, parkinsonian patients with FOG exhibit hesitancy and miss the action initiation window during Go/No-go tasks [54], indicating that attentional switching may be a crucial trigger for FOG. In clinical practice, behavioral self-management techniques are vital to alleviate the severity of FOG, applying specific auditory and visual cues to focus patient attention on gait [55], but the underlying mechanism is unknown. Our study indicates that persistent stimulation leads to a gradual decline in PPN-vGluT2 neuronal activity (as shown in Fig. S4C), suggesting that sustained attention may mitigate FOG through the glutamatergic circuit PPN. The mechanisms underlying this phenomenon warrant further investigation.

In summary, our study has elucidated the role of rostral PPN glutamatergic neurons in mediating multiple aspects of motor modulation through distinct neural circuits. These insights have clarified the previously debated function of excitatory neurons within the PPN, have enhanced our comprehension of the PPN’s mechanisms in motor regulation, and most critically, have offered pivotal indications for the development of novel DBS therapeutic approaches for patients with PD.

## Supplementary Information

Below is the link to the electronic supplementary material.

Supplementary file 1 (PDF 3316 KB)

Supplementary file 2 (WMV 1571 KB)

Supplementary file 3 (WMV 1071 KB)

Supplementary file 4 (WMV 21873 KB)

Supplementary file 5 (WMV 8082 KB)

Supplementary file 6 (WMV 32545 KB)

Supplementary file 7 (WMV 3364 KB)

Supplementary file 8 (WMV 10762 KB)

Supplementary file 9 (WMV 4027 KB)

Supplementary file 10 (WMV 2961 KB)
