# Supplementary material for: Glutamatergic Circuits in the Pedunculopontine Nucleus Modulate Multiple Motor Functions
Source: Neurosci Bull. 2024 Nov 11;40(11):1713–31. doi: 10.1007/s12264-024-01314-y (PMC11607253; doi:10.1007/s12264-024-01314-y)
Supplement: Supplementary file 1 — (PDF 3316 KB) [file 12264_2024_1314_MOESM1_ESM.pdf]

Supplemental Information

Figures S1- S10 and Figure Legends

Legends for Movies S1-S9

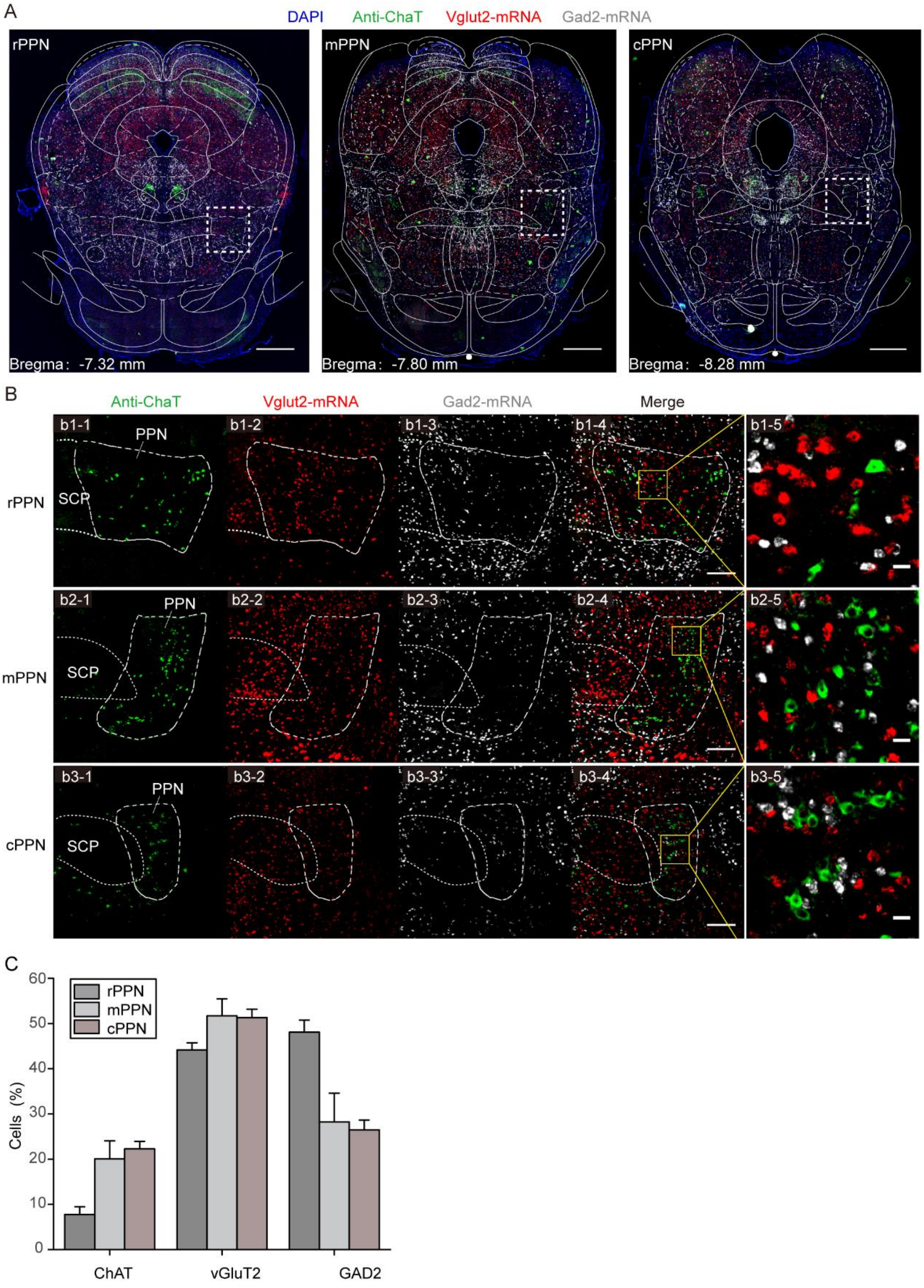

Fig. S1 Profile of the cell type in the PPN.

A The PPN is divided into three parts based on anterior-posterior coordinates: the rostral PPN (rPPN), the middle PPN (mPPN), and the caudal PPN (cPPN). Scale bars, 1000 μm.

B Multiplex fluorescent *in situ* hybridization and immunostaining reveal that ChAT (green), vGluT2 (red), and GAD2 (grey) neurons are mainly segregated in the rPPN (upper), mPPN (middle), and cPPN (lower). White dotted lines label the PPN region marked by ChAT immunostaining. Scale bars, 200 μm (left), 20 μm (right). Representative regions of merged images are windowed and enlarged in the right-most column.

C The percentage of each cell type among the total number of all three cell types. The percentage of ChAT neurons is low in the rPPN (~8%) and increases to ~22% in the caudal PPN. The percentage of vGluT2 neurons is ~44% in the rPPN and 51% in the cPPN. The percentage of GAD2 neurons is ~48% in the rPPN and ~26% in the cPPN (n = 3). Data are presented as the mean ± SD.

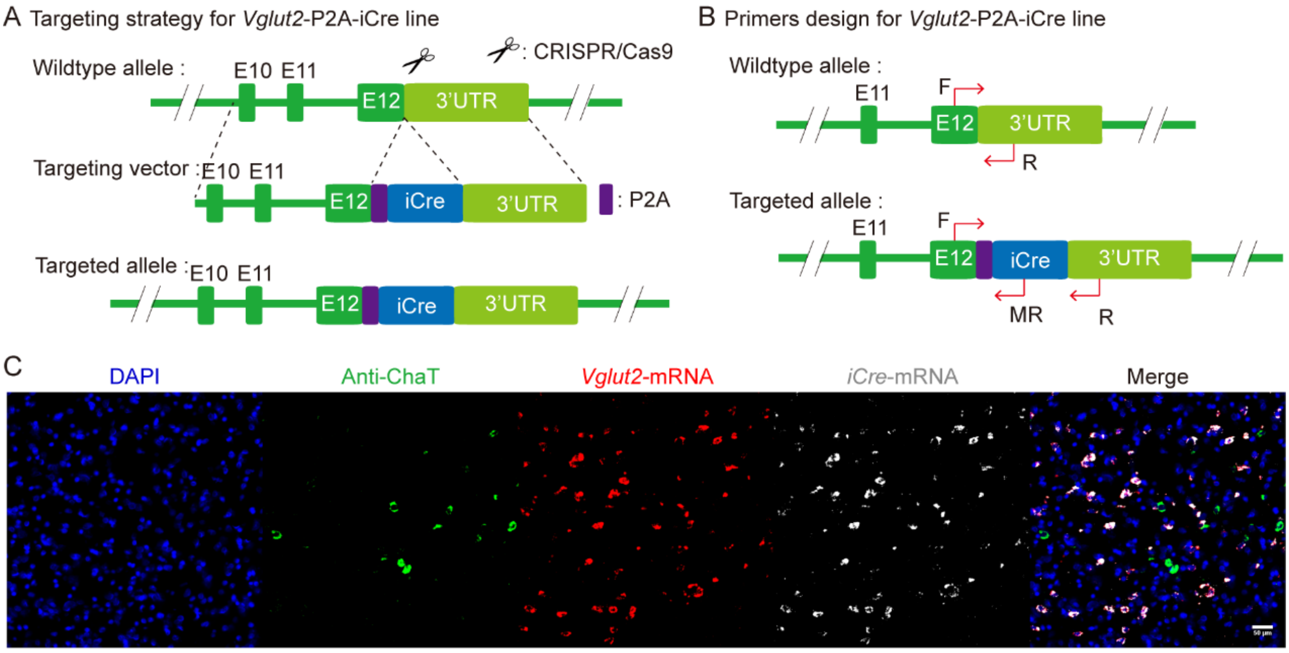

Fig. S2 Specificity assessment of iCre expression in vGluT2 neurons of the *Vglut2-P2A-iCre* rat line.

A Schematic of the *Vglut2-P2A-iCre* rat genetic construct. The *P2A-iCre* is engineered to knock in at the stop codon locus of the *Vglut2* gene, effectively replacing it.

B Primer design strategy for positive identification of the *Vglut2-P2A-iCre* rat line.

C Confirmation of iCre specificity in PPN-vGluT2 neurons. Green, anti-Chat by immunohistochemistry; red, detection of *Vglut2*-mRNA by RNAscope; grey, detection of *iCre*-mRNA by RNAscope. Scale bar, 50 μm.

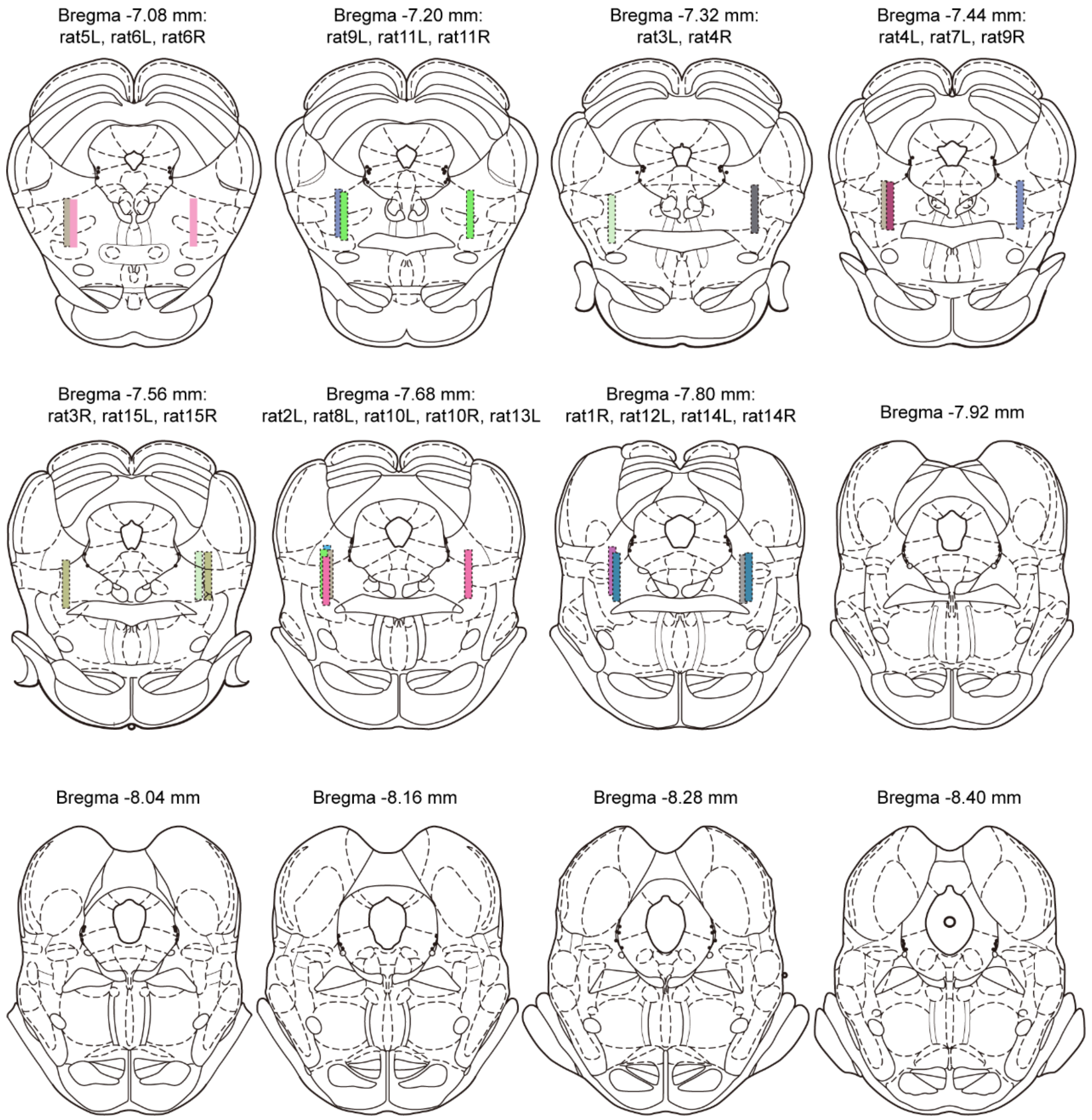

Fig. S3 Histologically verified positions of fiber placement and virus injection sites with GCaMP6s expression across all rats in the study. The representative diagram showing the recording sites for the activity of PPN-vGluT2 neurons is focused on the rPPN and mPPN.

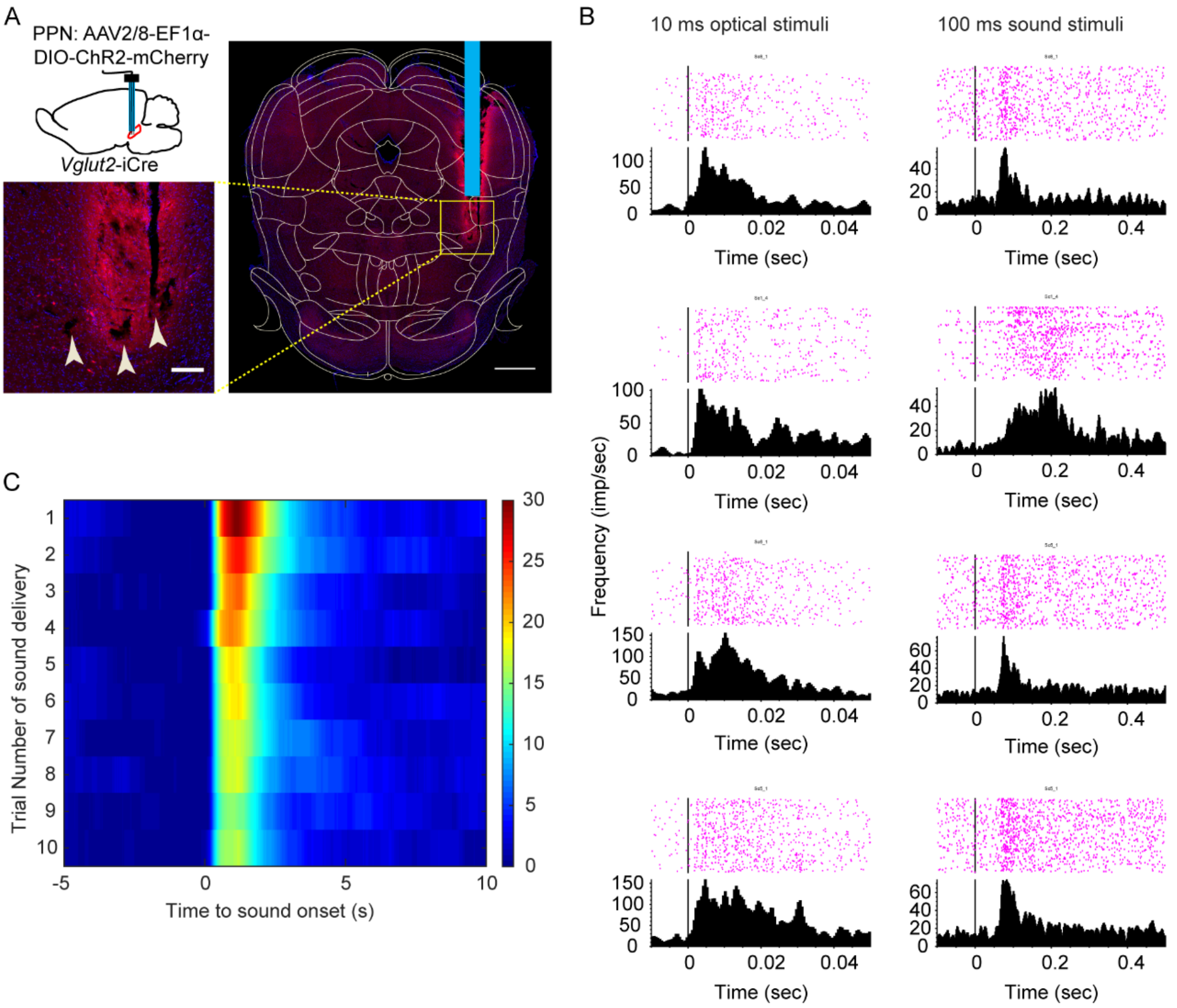

Fig. S4 PPN-vGluT2 neurons respond to acoustical stimulation.

A Representative image showing the position of the optical electrode. Scale bar in right panel, 1000 μm; scale bar in left lower panel, 200 μm.

B Four example neurons showing the labeled PPN-vGluT2 neurons response to the acoustical stimuli. Each row shows an example neuron.

C Heatmap of the PPN-vGluT2 neuronal Ca^{2+} signal in response to salient sound stimulation, according to the sequence of sound-delivery.

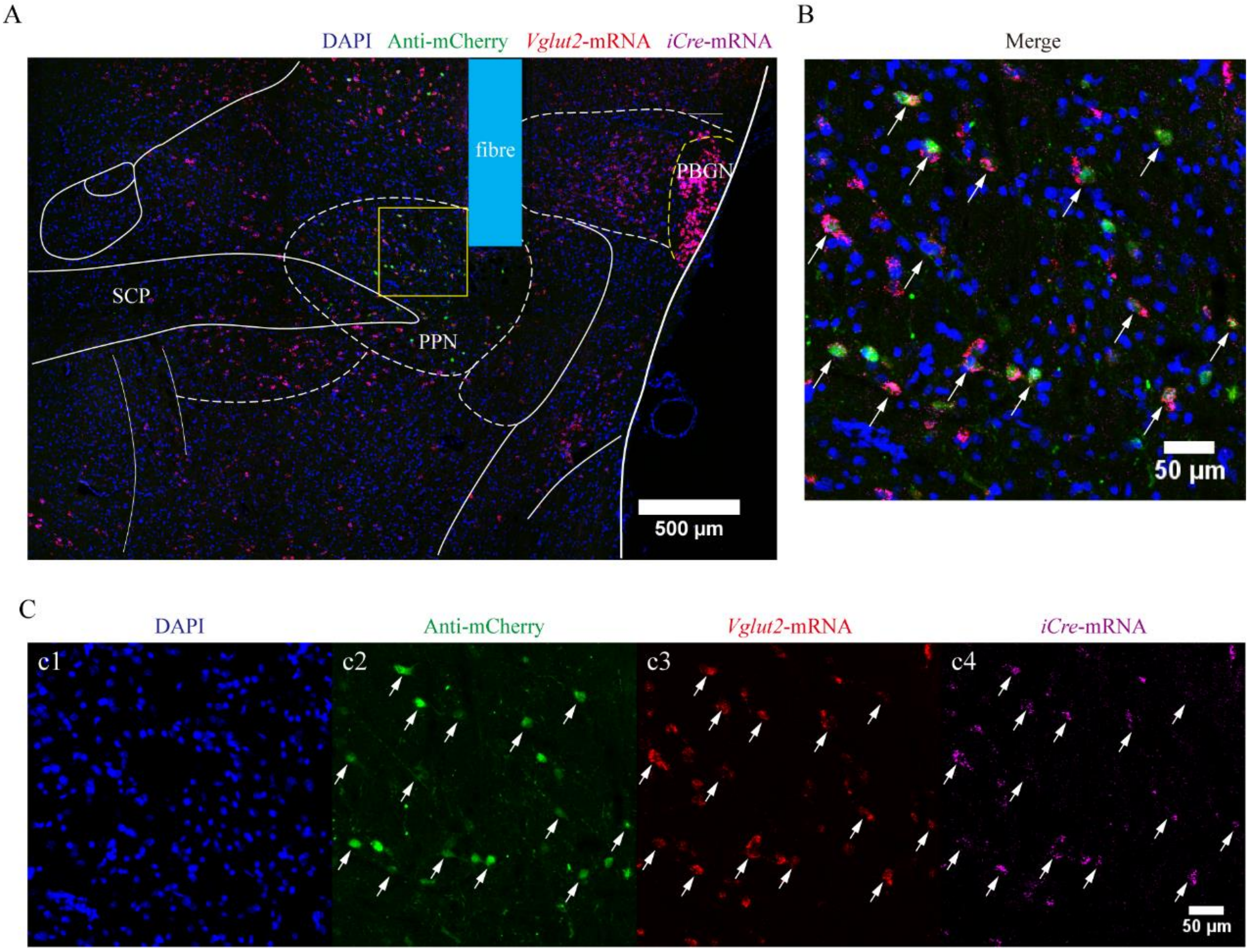

Fig. S5 Identification of the specificity of ChR2 expression in PPN-vGluT2 neurons.

A Representative image showing the specificity of ChR2 expression in PPN-vGluT2 neurons. Green, anti-mCherry by immunohistochemistry, as the marker for ChR2-labeled neurons; red, anti-*VgluT2*-mRNA by RNAscope; pink, anti-*iCre*-mRNA by RNAscope. Scale bar, 500 μm.

B, C Representative histology showing the expression of ChR2 in PPN-vGluT2 neurons. Scale bars, 50 μm.

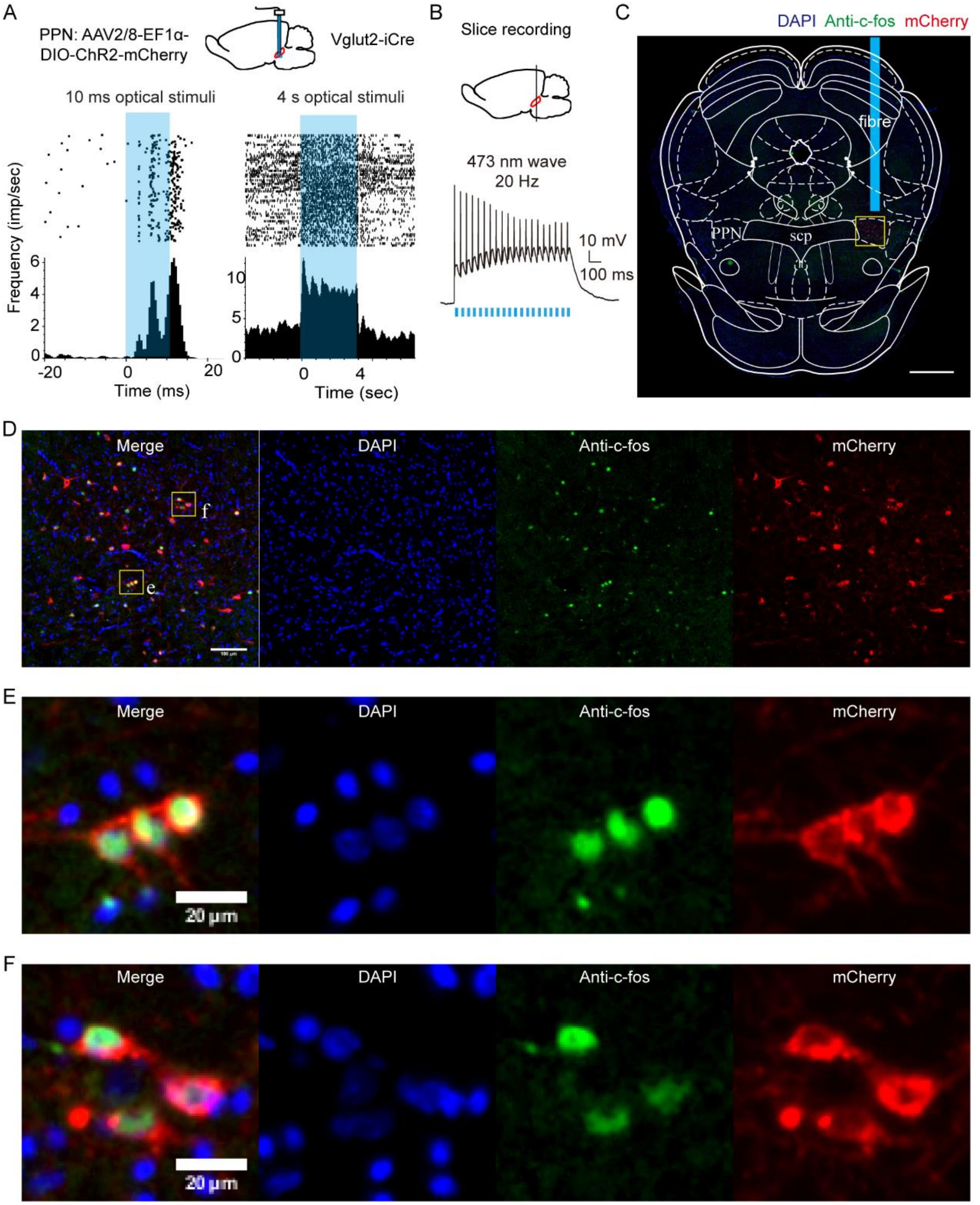

Fig. S6 The effectiveness of optogenetic activation of PPN-vGluT2 neurons, both *in vivo* and *in vitro*.

A An example neuron showing the ChR2-labeled PPN-vGluT2 neuronal response to 4-s optogenetic stimulation *in vivo*. Left panel, peristimulus time histogram aligned to 10-ms laser stimulation, which identifies the PPN-vGluT2 neuron labeled with ChR2. Right panel, histogram aligned to 4-s

laser stimulation, which reveals the firing pattern of the labeled PPN-vGluT2 neuron under experiment conditions.

B Whole-cell patch clamp recording from a representative neuron in an acute *in vitro* slice showing activation during optogenetic stimulation.

C-F Representative histology of c-fos immunostaining after optogenetic stimulation in the PPN. Red, ChR2-mCherry. Green, anti-c-fos. Scale bar in **C**, 1000 μm; **D** Window from **C**, scale bar, 100 μm; **E, F**, Window from **D**, scale bar, 20 μm.

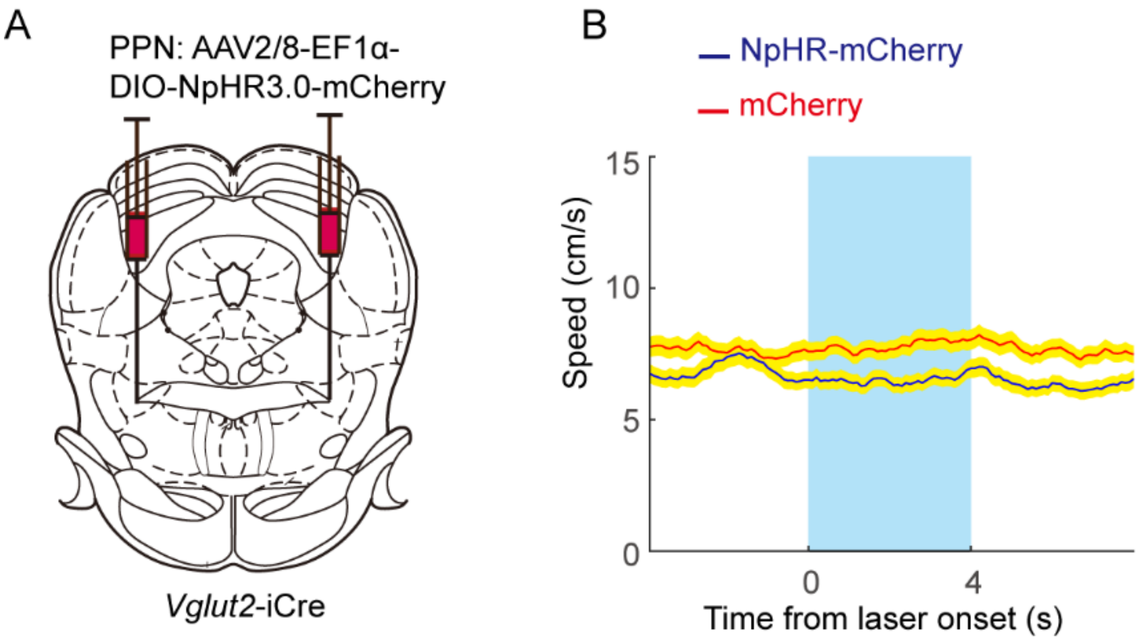

Fig. S7 Inhibition of PPN-vGluT2 neurons does not influence locomotion.

A Schematic of virus injection with halorhodopsin (NpHR) expression in PPN-vGluT2 neurons.

B Changes in averaged speed during locomotion induced by optogenetic inhibition of PPN-vGluT2 neurons. Blue line, experimental group with NpHR expression, *n* = 7; red line, control group with mCherry expression, *n* = 6. Data are presented as the mean ± SEM.

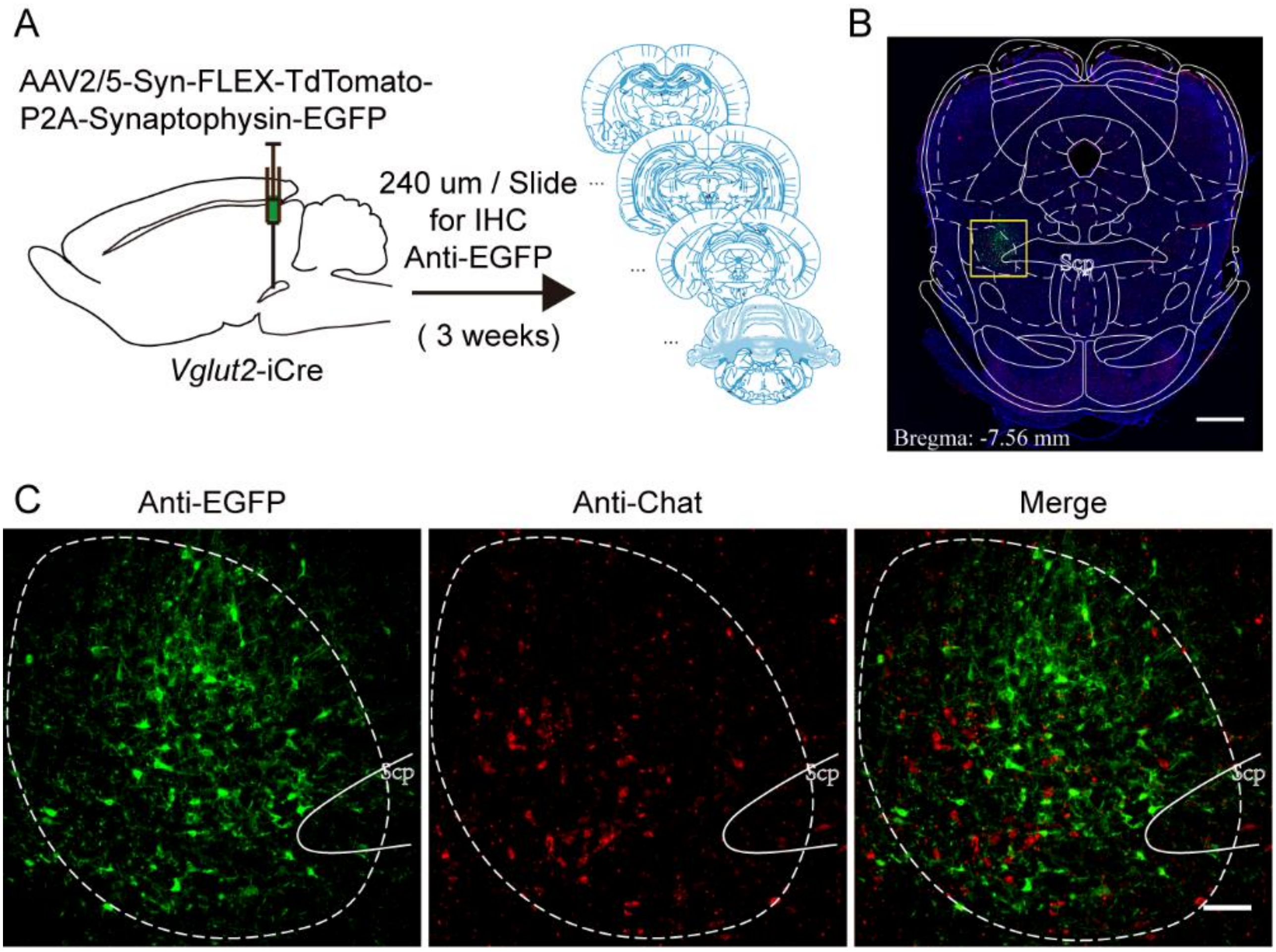

Fig. S8 Representative histology showing the viral injection site for the study of downstream outputs of PPN vGluT2 neurons.

A Schematic of labeling the terminals of PPN-vGluT2 neurons with the viral expression of EGFP-tagged synaptophysin (a marker for pre-synapses). The signals of EGFP labeling the terminals of PPN-vGluT2 neurons are amplified by immunohistochemistry.

B, C Representative histology showing viral injection site with EGFP expression specifically in the PPN. **C**, Region of interest is windowed from **B** and enlarged. Scale bar in **B**, 1000 μm; scale bar in **C**, 120 μm.

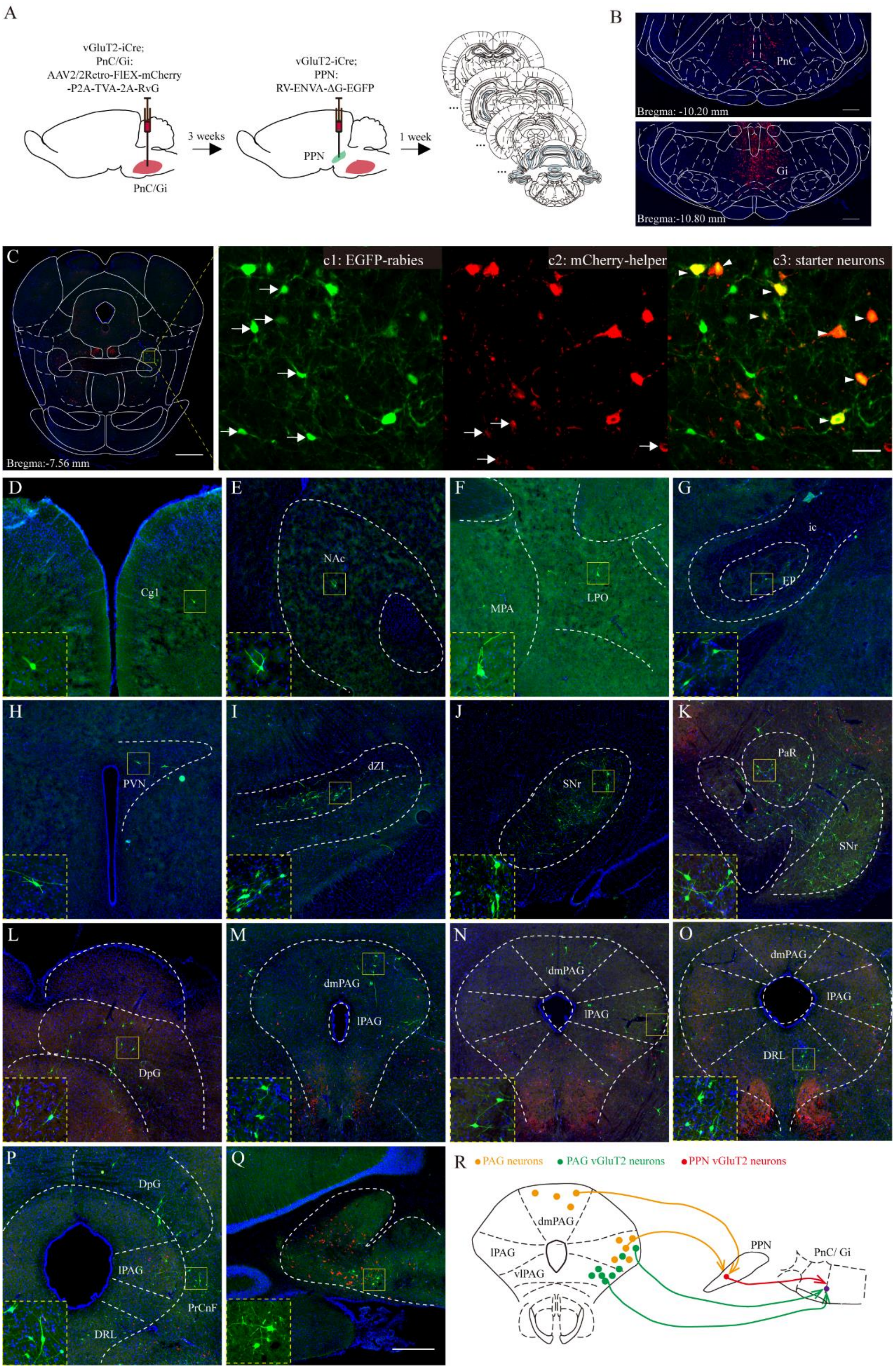
A
vGluT2-iCre;
PnC/Gi:
AAV2/2Retro-FlEX-mCherry
-P2A-TVA-2A-RvG
3 weeks
vGluT2-iCre;
PPN:
RV-ENVA-ΔG-EGFP
1 week
PPN
PnC/Gi
B
PnC
Bregma: -10.20 mm
Gi
Bregma:-10.80 mm
C
Bregma:-7.56 mm
c1: EGFP-rabies
c2: mCherry-helper
c3: starter neurons
D
Cg1
E
NAc
F
MPA
LPO
G
ic
EP
H
PVN
I
dZI
J
SNr
K
PaR
SNr
L
DpG
M
dmPAG
lPAG
N
dmPAG
lPAG
O
dmPAG
lPAG
DRL
P
DpG
lPAG
PrCnF
DRL
Q
R
PAG neurons
PAG vGluT2 neurons
PPN vGluT2 neurons
dmPAG
lPAG
vlPAG
PPN
PnC/ Gi

Fig. S9 Inputs of PPN-vGluT2 neurons projecting to the PnC/GiA.

A Schematic of upstream anatomical studies of PnC/GiA-projecting PPN-vGluT2 neurons.

B Representative histology showing the injection site of helper virus. Scale bar, 1000 μm.

C Representative histology showing the start cells of PnC/GiA-projecting PPN-vGluT2 neurons for retrograde trans-synaptic tracing. Scale bar, left panel: 1000 μm; scale bar, right panel: 50 μm.

D-Q Representative histology of inputs of PnC/GiA-projecting PPN-vGluT2 neurons: the ACC, NAc, MPA, LPO, GPi, PVN, ZI, SNr, PaR, SC, PAG, DRN, and Med. Scale bar, 400 μm.

R Schematic of the connections of the PAG, PPN, and PnC/GiA. The PAG vGluT2 neurons (green), which directly project to the PnC/GiA, differ from the PAG neurons (yellow), which are upstream of PnC/GiA-projecting PPN vGluT2 neurons (red).

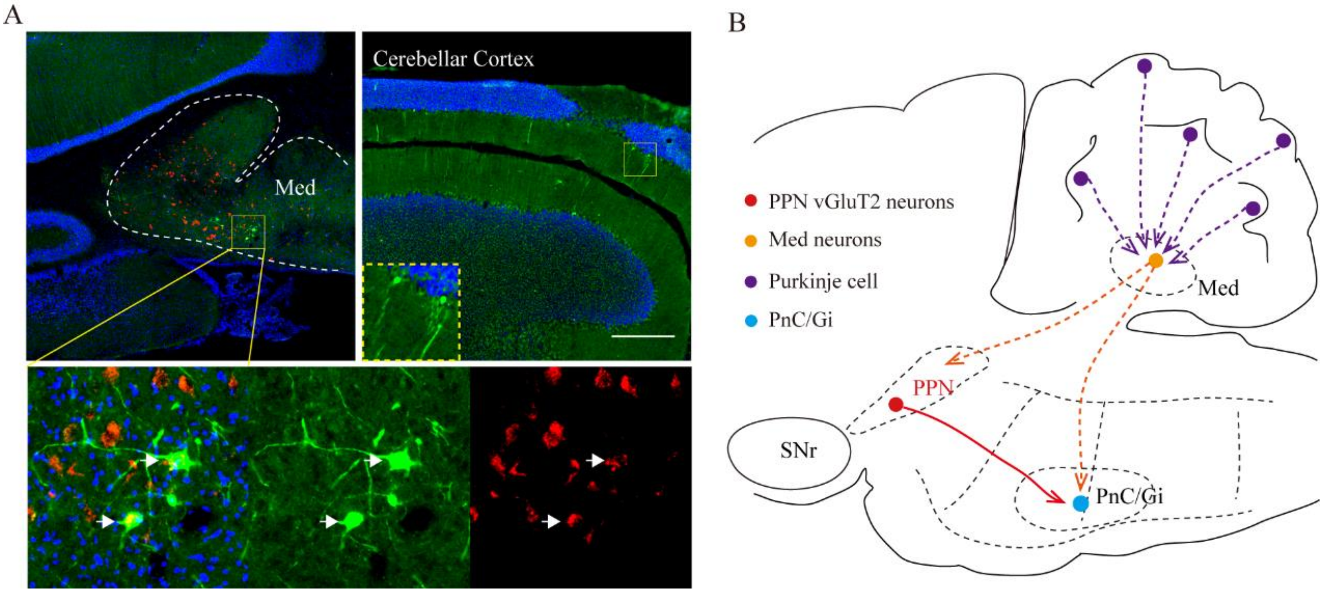

Fig. S10 Connections between PPN-vGluT2 neurons and the cerebellum.

A Representative histology showing inputs of PnC/GiA-projecting PPN-vGluT2 neurons in the cerebellum. Upper left panel, as in Fig. S11 Q, red shows the Med vGluT2 neurons retrogradely traced from the PnC/GiA; green shows the Med neurons retrograde transsynaptic tracing from PnC/GiA-projecting PPN-vGluT2 neurons; yellow shows the Med vGluT2 neurons with the ability to be start cells for retrograde transsynaptic tracing. Upper right panel, Purkinje cells are upstream of the yellow Med vGluT2 neurons. Scale bar in the upper panel, 400 μm

B Schematic of the connections of the PPN, PnC/GiA, and the cerebellum. The Med vGluT2 neurons are the relay nucleus for the connection between Purkinje cells and the PPN.

Movie S1. The activity of PPN vGluT2 neuronal responses to 100 ms of sudden sound stimulation.

Movie S2. Activation of rPPN vGluT2 neurons terminates locomotion.

Movie S3. Movements during walking, grooming, and eating are halted by optogenetic activation of rPPN vGluT2 neurons.

Movie S4. Movements during rearing, climbing, and balancing are halted by optogenetic activation of rPPN vGluT2 neurons.

Movie S5. Maintaining balance lasts for up to 20 s when motion is halted by optogenetic activation of rPPN vGluT2 neurons.

Movie S6. Swimming is halted by optogenetic activation of rPPN vGluT2 neurons, and rats maintain a stationary posture while they sink.

Movie S7. Respiration, reflected by the fluctuations of the abdomen, decreases remarkably and ceases completely during optogenetic activation of rPPN vGluT2 neurons.

Movie S8. Movements are halted by optogenetic activation of the terminals of PPN vGluT2 neurons projecting to the PnC/GiA.

Movie S9. Locomotion is promoted by optogenetic activation of the terminals of PPN vGluT2 neurons projecting to the ZI.
